# Safety and Efficacy of PTH 1‐34 and 1‐84 Therapy in Chronic Hypoparathyroidism: A Meta‐Analysis of Prospective Trials

**DOI:** 10.1002/jbmr.4566

**Published:** 2022-05-20

**Authors:** Giulia Puliani, Valeria Hasenmajer, Ilaria Simonelli, Valentina Sada, Riccardo Pofi, Marianna Minnetti, Alessia Cozzolino, Nicola Napoli, Patrizio Pasqualetti, Daniele Gianfrilli, Andrea M. Isidori

**Affiliations:** ^1^ Department of Experimental Medicine Sapienza University of Rome Rome Italy; ^2^ Oncological Endocrinology Unit Istituto di Ricovero e Cura a Carattere Scientifico (IRCCS) Regina Elena National Cancer Institute Rome Italy; ^3^ Service of Medical Statistics and Information Technology Fatebenefratelli Foundation of Health Research and Education Rome Italy; ^4^ Department of Biomedicine and Prevention University of Rome Tor Vergata Rome Italy; ^5^ Operative Research Unit of Osteo‐metabolic and thyroid diseases Fondazione Policlinico Universitario Campus Bio‐Medico Rome Italy; ^6^ Division of Bone and Mineral Diseases Washington University in St. Louis St. Louis MO USA; ^7^ Department of Public Health and Infectious Diseases, Section of Medical Statistics Sapienza University of Rome Rome Italy

**Keywords:** HYPOPARATHYROIDISM, PTH REPLACEMENT THERAPY, TERIPARATIDE, PTH1‐84, PTH1‐34

## Abstract

Hypoparathyroidism is the only endocrine deficiency for which hormone replacement therapy is not the standard of care. Although conventional treatments may control hypocalcaemia, other complications such as hyperphosphatemia, kidney stones, peripheral calcifications, and bone disease remain unmet needs. This meta‐analysis (PROSPERO registration number CRD42019126881) aims to evaluate and compare the efficacy and safety of PTH_1−34_ and PTH_1−84_ in restoring calcium metabolism in chronic hypoparathyroidism. EMBASE, PubMed, and CENTRAL databases were searched for randomized clinical trials or prospective studies published between January 1996 and March 2021. English‐language trials reporting data on replacement with PTH_1−34_ or PTH_1−84_ in chronic hypoparathyroidism were selected. Three authors extracted outcomes, one author performed quality control, all assessed the risk of biases. Overall, data from 25 studies on 588 patients were analyzed. PTH therapy had a neutral effect on calcium levels, while lowering serum phosphate (−0.21 mmol/L; 95% confidence interval [CI], −0.31 to −0.11 mmol/L; *p* < 0.001) and urinary calcium excretion (−1.21 mmol/24 h; 95% CI, −2.03 to −0.41 mmol/24 h; *p* = 0.003). Calcium phosphate product decreased under PTH_1−84_ therapy only. Both treatments enabled a significant reduction in calcium and calcitriol supplementation. PTH therapy increased bone turnover markers and lumbar spine mineral density. Quality of life improved and there was no difference in the safety profile between PTH and conventionally treated patients. Results for most outcomes were similar for the two treatments. Limitations of the study included considerable population overlap between the reports, incomplete data, and heterogeneity in the protocol design. In conclusion, the meta‐analysis of data from the largest collection to date of hypoparathyroid patients shows that PTH therapy is safe, well‐tolerated, and effective in normalizing serum phosphate and urinary calcium excretion, as well as enabling a reduction in calcium and vitamin D use and improving quality of life. © 2022 The Authors. *Journal of Bone and Mineral Research* published by Wiley Periodicals LLC on behalf of American Society for Bone and Mineral Research (ASBMR).

## Introduction

Hypoparathyroidism is a rare condition caused by defective release of parathyroid hormone (PTH), resulting in impaired mineral metabolism. The combination of a lower 25‐hydroxyvitamin D (25(OH)D) conversion rate and reduced calcium reabsorption and phosphate excretion from the renal tubules leads to hypocalcaemia, hyperphosphatemia, and hypercalciuria, whereas the lack of PTH itself results in reduced bone turnover and bone disease.^(^ ^1^ ^)^ Conventional therapy is based on supplementation with oral calcium salts and active vitamin D, but this often fails to normalize biochemical parameters other than serum calcium. Although the latter can be maintained at target levels in most patients, the failure to control serum phosphate levels, calcium phosphate product, and calcium and phosphate excretion increases the risk of peripheral calcifications, kidney stone formation, kidney infections,^(^ ^2^, ^3^ ^)^ and, ultimately, impaired kidney function.^(^ ^4^ ^)^

Over the last two decades, several attempts have been made to develop a physiological replacement therapy for hypoparathyroidism, the only endocrine deficiency for which replacement with the missing hormone is not currently the standard of care. PTH deficiency is now known to have significant effects on patients' quality of life (QoL),^(^ ^5^ ^)^ morbidity,^(^ ^6^, ^7^, ^8^ ^)^ and mortality,^(^ ^8^ ^)^ meaning that such a therapy is urgently needed.

Human PTH_1−34_, teriparatide, is a fragment of the natural molecule widely used to treat osteoporosis^(^ ^9^ ^)^ that has been also used in hypoparathyroidism since 1996.^(^ ^10^ ^)^ Its short half‐life necessitates multiple daily injections, reducing tolerability. Later, the more stable recombinant human PTH_1−84_ has been approved for the treatment of hypoparathyroidism.^(^ ^11^ ^)^ Nevertheless, as hypoparathyroidism is a rare disease with few controlled trials, and PTH has multiple systemic targets, the management and safety of PTH replacement therapy—and the ideal candidates for such a therapy—have yet to be established.^(^ ^12^ ^)^

To our knowledge, only two other meta‐analyses have investigated some of the aspects related to PTH replacement therapy. These both limited their analysis to randomized controlled trials (RCTs) (four and five articles, respectively).^(^ ^13^, ^14^ ^)^ Liu and colleagues^(^ ^14^ ^)^ included only data on biochemical parameters, whereas Palui and colleagues^(^ ^13^ ^)^ did not compare PTH_1−34_ and PTH_1−84_.

RCTs undoubtedly represent the highest level of evidence. However, open‐label and uncontrolled studies may still provide valuable evidence, especially in rare diseases, in which the small sample size and logistic limitations often render RCTs unfeasible or biased. Furthermore, the inclusion of all prospective studies, including real‐world ones, in a systematic review and meta‐analysis increases the power of the analysis, reduces publication bias, better reflects real‐life settings.^(^ ^15^, ^16^ ^)^ In fact, the European Medicine Agency's guidelines state that study designs other than RCTs (prospective open‐label or crossover studies) are acceptable for rare conditions and clinical trials in small populations, if they help to improve the interpretability of the study results.^(^ ^17^ ^)^

For all these reasons, we performed a meta‐analysis of both RCTs and nonrandomized prospective studies to investigate the effects of PTH_1−34_ and PTH_1−84_ on serum and urinary calcium and phosphate levels, serum vitamin D levels, doses of calcium and active vitamin D supplements, bone markers, bone mineral density, QoL, and safety in patients with chronic hypoparathyroidism.

## Materials and Methods

This study was performed in accordance with the Preferred Reporting Items for Systematic reviews and Meta‐Analyses (PRISMA) statement^(^ ^18^ ^)^ and registered on PROSPERO (CRD42019126881; https://www.crd.york.ac.uk/prospero/display_record.php?RecordID=126881).

### Data sources and searches

Between February and October 2019, we searched EMBASE, PubMed, and Cochrane Central Register of Controlled Trials (CENTRAL) databases for articles published since 1995. The following search terms were used: “Hypoparathyroidism AND PTH”; “Hypoparathyroidism AND hPTH”; “Hypoparathyroidism AND rhPTH”; “Hypoparathyroidism AND parathyroid hormone”; “Hypoparathyroidism AND teriparatide”; “Hypoparathyroidism AND replacement therapy”. A search update was conducted in March 2021 and two additional studies were included.^(^ ^19^, ^20^ ^)^

### Study selection

Eligibility criteria: (i) RCTs and open‐label prospective trials; (ii) PTH therapy (1‐34 or 1‐84) administered to children and adults with chronic hypoparathyroidism; and (iii) studies reporting at least one of the outcomes of interest. Three authors (GP, VH, and VS) independently screened all articles by title and abstracts. Full‐text articles from eligible papers were retrieved and analyzed. Disagreements were solved by consensus after open discussion.

### Outcomes

Studies had to report the effect of treatment on at least one of the following: biochemical parameters, supplementation (calcium and calcitriol or equivalents), bone mineral density (lumbar spine, total hip, femoral neck, distal radius), or QoL and/or adverse events.

### Data extraction and quality assessment

Three authors (GP, VH, and VS) independently extracted the following data: study design, study duration, age, sex, etiology and duration of hypoparathyroidism, treatment schedule, study objectives, and outcome results. The quality control checks on extracted data were performed by another investigator (IS). For overlapping populations, data were only extracted from the most recent and complete study. For one cohort, two recent long‐term studies were excluded due to significant dropout.^(^ ^21^, ^22^ ^)^ Where there was a crossover between different dose regimens (once versus twice daily administration of PTH_1−34_^(^ ^23^, ^24^ ^)^ or continuous infusion versus twice daily administration of PTH_1−34_^(^ ^25^, ^26^ ^)^), data from the most commonly used regimen were extracted to avoid patient duplication. For extension studies or studies enrolling patients previously treated with PTH, we only extracted data if new baseline parameters were provided.

For continuous outcomes, mean and standard deviation (SD) were extracted when available. Alternatively, median and range or interquartile range were used. Baseline values (for conventional therapy) and absolute or percentage change from baseline were extracted as reported. SDs were calculated when only standard errors (SEs) were reported. When median and range (or interquartile range) were reported, mean and SD were estimated.^(^ ^27^ ^)^ Visual inspection of publication bias was performed by funnel plot (available in Appendix S1), and when there were enough studies Egger's test was also performed. Quality of studies was assessed by Jadad scale^(^ ^28^ ^)^ for RCTs and methodological index for nonrandomized studies (minors) score^(^ ^29^ ^)^ for uncontrolled trials.

### Data synthesis and statistical analysis

To pool continuous data (mean and SD), the mean difference (MD) between post‐values and pre‐values was calculated according to a random effect model. The SE of the MD was calculated according to Cochrane,^(^ ^30^ ^)^ assuming a pre‐post correlation of 0.5. The sensitivity analysis was performed assuming a correlation of 0.2 or 0.7 (results available in Appendix S1). MD was reported with a 95% confidence interval (CI). To pool QoL data (Physical Composite Score [PCS] and Mental Composite Score [MCS]), the standardized mean difference (SMD) between post‐values and pre‐values and the corresponding SE were calculated.

When a meta‐analysis could not be performed, descriptive information on the magnitude of the effect was provided in terms of median and range (minimum–maximum) and plotted as a bubble chart, with bubble size corresponding to study size. In addition, the weighted average for pretreatment and posttreatment values and absolute and percentage change was calculated using sample size as the weight. Weighted averages are reported with SE and are available in Appendix S1.

Heterogeneity was evaluated with forest plot visual inspection of CI overlap and outliers and quantified by *I* ^2^ index. The heterogeneity was classified as moderate if *I* ^2^ was at least 50%, and high for *I* ^2^ of 75% or more. Subgroup analysis was performed by stratifying the studies by type of treatment (PTH_1−34_, PTH_1−84_) when possible. Additional subgroup analyses were performed for RCTs only, according to supplement withdrawal protocols for serum and urinary calcium, for study duration (<1 year or ≥1 year) and for pediatric compared to adult patients. The difference between the group‐specific overall effect sizes was investigated by applying a test of between‐group differences based on the Qb statistic.

Data on adverse events were presented as an overall percentage and SE. When data were also available for controls, a meta‐analysis for binary data was performed using relative risk (RR) as effect size. A *p* value <0.05 was considered statistically significant. Data were analyzed with the statistical software package STATA v16 (StataCorp, LLC, College Station, TX, USA).

## Results

### Study selection

**Fig. 1:**
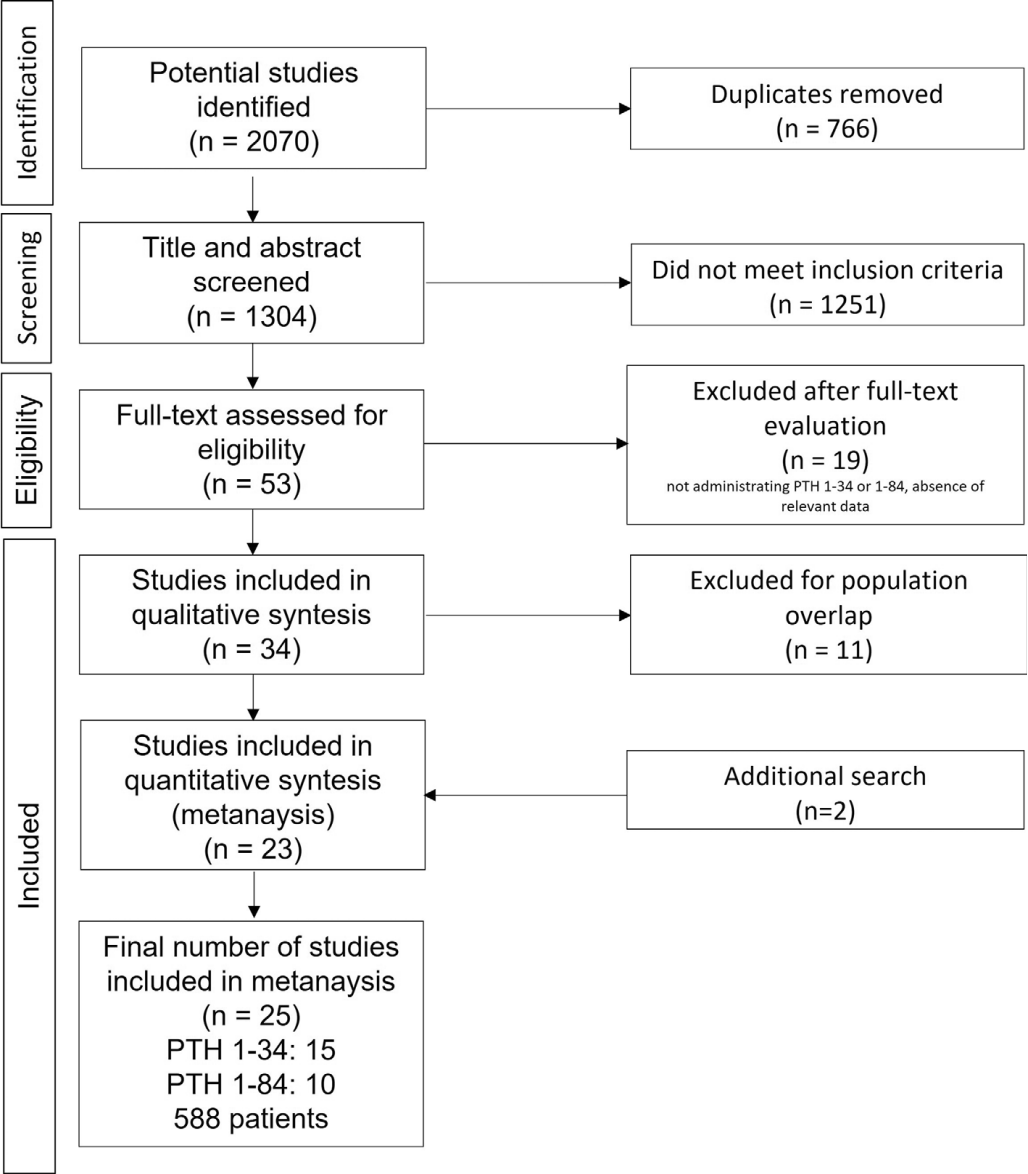
Flowchart.

The original literature search retrieved 2070 studies. Of these, 2017 were excluded after abstract screening (1251) or because they were duplicates (766), and 19 after full‐text evaluation. Ultimately, 34 studies were included, and two further studies were added after the later search performed in March 2021, as reported in Fig. 1. Thirty‐six studies (15 on PTH_1−34_ and 21 on PTH_1−84_) were therefore analyzed. They comprised seven RCTs, 23 open‐label trials, four crossover trials, one self‐controlled trial, and one randomized, dose‐blinded, fixed‐dose trial. Their main characteristics are summarized in Table 1 (RCTs) and Table 2 (non‐RCTs).

**Table 1:** Controlled Trials on PTH_1−34_ and PTH_1−84_

First author, year, reference	Study design	Center	Total number of patients (PTH/controls) ITT^a^	Age (years)	HypoPTH etiology	Disease duration	Drug	Dosage and regimen	Study duration (months)	Study outcome(s)^b^	Quality (Jadad)	Used for meta‐analysis	Sponsored
Harslof, 2015^(^ ^69^ ^)^	RCT	Aarhus (Denmark)	62 (32/30); available: 28/30	Range, 31–78	NA	–	1‐84 versus pl	100 μg/day	6	Biochemistry Body composition Metabolic evaluation	5 (high)	No	Yes
Sikjaer, 2014^c^ ^(^ ^42^ ^)^	RCT	Aarhus (Denmark)	62 (32/30)	Range, 31–78	PS: 48/62 I: 4/62	8.0 (1–37)^d^ (pl) 9.5 (2–33)^d^ (PTH)	1‐84 versus pl	100 μg/day	6	**Biochemistry** Muscle function Postural stability QOL	5 (high)	Yes	Yes
Sikjaer, 2013^c^ ^(^ ^70^ ^)^	RCT	Aarhus (Denmark)	62 (32/30); available 21/17	Range, 31–78	PS: 37/38 I: 1/38	6 (2–32)^d^ (pl) 7 (3–34)^d^ (PTH)	1‐84 versus pl	100 μg/day	6	Biochemistry ECG	5 (high)	No	Yes
Sikjaer, 2011^c^ ^(^ ^43^ ^)^	RCT	Aarhus (Denmark)	62 (32/30)	Range, 31–78	PS: 48/62 I: 4/62	8.0 (1–37)^d^ (pl) 9.5 (2–33)^d^ (PTH)	1‐84 versus pl	100 μg/day	6	**Calcium and calcitriol supplementation** Biochemistry BMD **Adverse events**	5 (high)	Yes	Yes
Vokes, 2018^e^ ^(^ ^47^ ^)^	RCT	Multicentre (same as Replace Study)	122 (83/39)	49.5 ± 13.3 (pl) 46.6 ± 12.3 (PTH) (mean ± SD)	PS: 28/39 (pl) 59/83 (PTH)	14.6 ± 11.2 (PTH) 11.8 ± 8.1 (pl)	1‐84 versus pl	50–100 μg/day	6	**QOL**	5 (high)	No	Yes
Clarke, 2016^e^ ^(^ ^39^ ^)^	RCT	Multicentre (Replace Study)	124 (84/40)	48,9 (21‐73)^c^ (pl) 46.6 (19‐74)^c^ (PTH) (mean ± SD)	–	11.6 (2–38)^d^ (pl) 14.6 (2–50)^d^ (PTH)	1‐84 versus pl	50–100 μg/day	6	**Biochemistry**	5 (high)	Yes	Yes
Mannstadt, 2013^e^ ^(^ ^11^ ^)^	RCT	Multicentre (Replace Study)	134 (90/44)	48.5 ± 13.7 (pl) 47.0 ± 12.2 (PTH) (mean ± SD)	PS: 31/44 (pl) 68/90 (PTH) I: 8/44 (pl) 14/90 (PTH) A: 4/44 (pl) 5/90 (PTH) G: 1/44 (pl) 2/90 (PTH)	14.1 ± 11.1 (PTH) 11.0 ± 8.0 (pl)	1‐84 versus pl	50–100 μg/day	6	Biochemistry **Calcium and calcitriol supplementation** **Adverse events**	5 (high)	Yes	Yes
Winer, 2003^(^ ^32^ ^)^	Randomized open‐label	NIH Bethesda	27 (14/13)	41 ± 15.4^f^ (mean ± SD)	PS: 41% I: 30% A: 7% G: 22%	15 ± 11.6^f^	1‐34 versus CRT	37 ± 2.6 (0.5 μg/kg/dose) twice daily	36	**Calcium and calcitriol supplementation** **Biochemistry** **BMD** **Adverse events**	2 (low)	Yes	No
Winer, 2010^(^ ^31^ ^)^	Randomized open‐label	NIH Bethesda	12 (7/5)	9.75 ± 2.73 (mean ± SD)	PS: 0 I: 5 A: 4 G: 1 NA: 2	NA	1‐34 versus CRT	0.6 μg/kg ± 0.5 twice daily	36	**Biochemistry** **Adverse events**	2 (low)	Yes	Yes
Winer, 1996^(^ ^10^ ^)^	Randomized crossover	NIH Bethesda	10	45.4 ± 14^f^ (mean ± SD)	PS: 40% I: 10% A: 20% G: 30%	17.8 ± 13.3^f^	1‐34	0.5–3 μg/kg once daily	15	**Calcium and calcitriol supplementation** **Biochemistry** **Adverse events**	1 (low)	Yes	No

A = autoimmune; BMD = bone mineral density; CRT = conventional replacement therapy; G = genetic; I = idiopathic; ITT = intention to treat; NA = not available; pl = placebo; PS = postsurgical; QOL = quality of life; RCT = randomized control trial.

^a^ Where a high percentage of patients did not complete the study, the number of patients with available data has also been reported.

^b^ Outcomes in bold were included in the meta‐analysis.

^c^ Same cohort as Harslof, 2015^(^ ^69^ ^)^.

^d^ Minimum–maximum range.

^e^ Same cohort as Vokes, 2018^(^ ^47^ ^)^.

^f^ The study provided individual patient data, and mean ± SD has been calculated.

**Table 2:** Uncontrolled Trials on PTH_1−34_ and PTH_1−84_

Author, year, reference	Study design	Center	Number of patients ITT	Age (years)	HypoPTH etiology	Duration of disease (years)	Drug	Dosage and regimen	Study duration (months)	Main outcome(s)^a^	Quality of study (minors)	Used for meta‐analysis	Sponsored
Rubin, 2016^(^ ^36^ ^)^	Open‐label	Columbia	33	47 ± 2.3 (mean ± SD)	PS: 20/33 A: 12/33 DGS: 1/33	17.4 ± 3	1‐84	100 μg every other day	72	**Calcium and calcitriol supplementation** **Biochemistry** **BMD** **Adverse events**	11	Yes	Yes
Cusano, 2013^b^ ^(^ ^71^ ^)^	Open‐label	Columbia	27	51 ± 12 (mean ± SD)	PS: 16/27 A: 10/27 DGS: 1/27	20 ± 15	1‐84	100 μg every other day	48	Calcium and calcitriol supplementation Biochemistry BMD Adverse events	10	No	Yes
Cipriani, 2018^b^ ^(^ ^72^ ^)^	Open‐label	Columbia	35	Pre‐M: 45.8 ± 11.8 (mean ± SD) Post‐M: 54 ± 9.8 (mean ± SD)	PS: 22/35 A, I: 12/35 DGS: 1/35	–	1‐84	100 μg every other day	18	BMD TBS	11	No	Yes
Tay, 2019^b^ ^(^ ^21^ ^)^	Open‐label	Columbia	24	46.2 ± 2.8 (mean ± SD)	PS: 13/24 A: 10/24 DGS: 1/24	29.9 ± 3.3	1‐84	100 μg every other day	96	Calcium and calcitriol supplementation Biochemistry BMD	12	No	Yes
Rubin, 2010^b^ ^(^ ^73^ ^)^	Open‐label	Columbia	33	48.2 ± 12 (mean ± SD)	PS: 18/33 A: 13/33 DGS: 2/33	17 ± 13	1‐84	100 μg every other day	24	Calcium and calcitriol supplementation Biochemistry BMD, qCT Histomorphometry	4	No	Yes
Rubin, 2010^b^ ^(^ ^74^ ^)^	Open‐label	Columbia	30	49 ± 12 (mean ± SD)	PS: 15/30 I: 11/30 A: 1/30 DGS: 2/30 G: 1/30	19 ± 15	1‐84	100 μg every other day	24	Calcium and calcitriol supplementation Biochemistry BMD	5	No	Yes
Cusano, 2013^b^ ^(^ ^75^ ^)^	Open‐label	Columbia	54	46 ± 14 (mean ± SD)	PS: 27 A: 26 DGS: 1	13 ± 12	1‐84	100 μg every other day	48	Biochemistry QOL	6	No	Yes
Cusano, 2014^b^ ^(^ ^46^ ^)^	Open‐label	Columbia	69	46 ± 2 (mean ± SD)	PS: 42/69 I: 26/69 DGS: 1/69	12 ± 1	1‐84	Starting dose: 100 μg every other day (range 25–100 μg daily)	60	Biochemistry **QOL**	9	Yes	Yes
Tabacco, 2019^b^ ^(^ ^22^ ^)^	Open‐label	Columbia	20	44.9 ± 3 (mean ± SD)	PS: 12/20 I: 8/20	25.7 ± 3	1‐84	Starting dose 100 μg/day every other day (range 25–75 μg daily)	96	Biochemistry QOL	11	No	Yes
Mannstadt, 2019^(^ ^40^ ^)^	Open‐label (RACE)	Multicentre (USA)	49	48.1 ± 9.78 (mean ± SD)	–	15.9 ± 12.5	1‐84	Starting dose 25 or 50 μg/day (range 25–100 μg/day)	60	**Calcium and calcitriol supplementation** **Biochemistry** **Adverse events** BMD	11	Yes	Yes
Upreti, 2017^(^ ^38^ ^)^	Open‐label	New Dehli	8	35.8 ± 6.8 (mean ± SD)	PS: 3/8 I: 5/8	2.1 (median)	1‐34	Starting dose 20 μg twice daily	18	**Calcium and calcitriol supplementation** **Biochemistry** QoL **BMD**	10	Yes	No
Misof, 2016^(^ ^76^ ^)^	Open‐label	NY, Columbia, Vienna	30	1‐year cohort 55 (41–61)^c^ 2‐year cohort 47 (40–60)^c^	PS: 19/30 A: 11/30	1‐year cohort 16 (5–40)^d^ 2‐year cohort 17 (5.5–28.5)^d^	1‐84	–	24	**Biochemistry** Histomorphometry	4	Yes	Yes
Gafni, 2018^(^ ^48^ ^)^	Open‐label	NIH	32	39.5 (range, 16–60)	PS: 20/32 G: 6/32 DGS: 2/32	>1	1‐34	0.40 μg/kg twice daily	60	Biochemistry Renal imaging **Adverse events**	9	Yes	Yes
Gafni, 2015^e^ ^(^ ^34^ ^)^	Open‐label	NIH	9	37 ± 13.3 (mean ± SD)	PS: 4/9 DGS: 2/9 G: 2/9 NA: 1/9	18.6 ± 18.4	1‐34	0.45 ± 0.09 μg/kg/day (in 2 doses), 0.37 ± 0.17 μg/kg/day (in 3 doses)	60 (20–60)	**Calcium and calcitriol supplementation** **Biochemistry** **Adverse events**	5	Yes	Yes
Gafni, 2012^e^ ^(^ ^49^ ^)^	Open‐label	NIH	5	28 ± 17.3 (mean ± SD)	PS: 2/5 G: 1/5 DGS: 1/5 I: 1/5	10.4 ± 6.7	1‐34	0.57 ± 0.24 μg/kg/day (in 2 or 3 doses)	18	Biochemistry **BMD** Histomorphometry	6	Yes	Yes
Winer, 2014^(^ ^26^ ^)^	Crossover	NIH	12	15.7 ± 0.96 (mean ± SE)	A: 5/12 G: 7/12	14.1 ± 0.9	1‐34	Twice daily (versus pump)^f^	6.5	**Calcium and calcitriol supplementation** **Biochemistry**	9	Yes	Yes
Winer, 2008^g^ ^(^ ^24^ ^)^	Crossover	NIH	14	9 ± 3.5 (mean ± SD)	PS: 1/14 I: 7/14 G: 1/14 A: 5/14	3.8 ± 3.3	1‐34	0.7 μg/kg/day (in 2 doses) (versus once daily)^f^	7	Calcium and calcitriol supplementation Biochemistry Adverse events	9	No	No
Winer, 1998^(^ ^23^ ^)^	Crossover	NIH	17	41 ± 13.9 (mean ± SD)	PS: 9/17 I: 1/17 G: 6/17 A: 1/17	19 ± 11.3	1‐34	0.7 μg/kg/day (in 2 doses) (versus once daily)^f^	3.5	**Calcium and calcitriol supplementation** **Biochemistry** **Adverse events**	7	Yes	No
Matarazzo, 2014^(^ ^33^ ^)^	Self‐controlled trial	Torino, Italy	6	9.8 ± 5.1 (mean ± SD)	A: 3/6 G: 1/6 DGS: 2/6	–	1‐34	0.7 ± 0.24 μg/kg/day (in 2 doses)	30	**Calcium and calcitriol supplementation** **Biochemistry** Renal imaging **Adverse events**	7	Yes	No
Bilezikian, 2017^(^ ^44^ ^)^	R, dose‐blinded, fixed‐dose (RELAY)	Columbia	42	48.4 ± 10.51 (mean ± SD)	–	–	1‐84	25–50 μg/day once daily	2	**Calcium and calcitriol supplementation** **Biochemistry** **Adverse events**	11	Yes	Yes
Lakatos, 2016^(^ ^37^ ^)^	Open‐label (REPEAT)	Hungary	24	52.7 ± 10.9 (mean ± SD)	PS: 20/24 I: 3/24 NA: 1/24	15.1 ± 12.6	1‐84	50 μg/day once daily	6	**Calcium and calcitriol supplementation** **Biochemistry** **Adverse events** **BMD**	12	Yes	Yes
Winer, 2012^(^ ^25^ ^)^	Open‐label, R, crossover	NIH	8	46 ± 5.6 (mean ± SD)	PS: 8/8	3.4 ± 2.3 years	1‐34	37 ± 14 μg/day (in 2 doses) (versus pump)^f^	6	**Biochemistry** **Adverse events** Muscle indexes	6	Yes	No
Santonati, 2015^(^ ^45^ ^)^	Open‐label	Multicentre (Italy)	42	55.8 ± 10.4 (mean ± SD)	PS: 42/42	7.3 ± 5.1 years	1‐34	20 μg twice daily	6	**Calcium and calcitriol supplementation** Biochemistry **Adverse events** **QoL**	9	Yes	No
Palermo, 2018^h^ ^(^ ^35^ ^)^	Open‐label	Multicentre (Italy)	42	55.8 ± 10.4 (mean ± SD)	PS: 42/42	–	1‐34	20 μg twice daily	24	**Calcium and calcitriol supplementation** **Biochemistry** **Adverse events** QoL	6	Yes	No
Marcucci, 2021^(^ ^19^ ^)^	Open‐label	Florence (Italy)	12	48.6 ± 18.4 (mean ± SD)	PS: 9/12 I: 3/12	12.6 years (range, 2–36)	1‐34	36 ± 8.4 μg daily (in 2 doses)	3	**Calcium and calcitriol supplementation** **Biochemistry** **Adverse events** **BMD**	8	Yes	No
Winer, 2018^(^ ^20^ ^)^	Open‐label	NIH Bethesda	14	10.5 ± 3.2 (mean ± SD)	A: 5/14 G: 9/14	8.57 ± 4.3	1‐34	0.5 μg/kg/day (in 2 doses)	6.9 (1.5–10) years	**Biochemistry** **Adverse events** Linear growth Bone mineral accrual Cerebral calcifications	6	Yes	Yes

A = autoimmune; BMD = bone mineral density; G = genetic; I = idiopathic; ITT = intention to treat; OG = other genes; Post‐M = postmenopausal; Pre‐M = premenopausal; PS = postsurgical; R = randomized.

^a^ Outcomes in bold were included in the meta‐analysis.

^b^ Same cohort as Rubin, 2016^(^ ^36^ ^)^.

^c^ Median and 25th–75th percentile.

^d^ Minimum–maximum range.

^e^ Same cohort as Gafni, 2018^(^ ^48^ ^)^.

^f^ Data not extracted for meta‐analysis.

^g^ Significant overlap with Winer, 2014^(^ ^26^ ^)^.

^h^ Same cohort as Santonati, 2015^(^ ^45^ ^)^.

Twenty‐six studies declared funding by pharmaceutical companies. PTH_1−34_ daily dose ranged from 20 to 40 μg in adults and from 0.4 to 1.2 μg/kg daily in children, mostly administered twice daily. The PTH_1−84_ daily dose ranged from 25 to 100 μg, administered once daily or every other day. In 11 studies, the populations partially overlapped, so the studies were excluded to avoid duplications. The participants' age ranged from 7 to 78 years. A total of 242 patients (183 females and 62 males) were enrolled in RCTs, and 343 patients (262 females and 81 males) in non‐RCTs. When reported, the most frequent etiologies of hypoparathyroidism were postsurgical (281/401 patients, 70%) followed by idiopathic, autoimmune (polyglandular failure), and genetic causes (DiGeorge syndrome; hypoparathyroidism, deafness, and renal disease syndrome; calcium sensing receptor activating mutations), as reported in Tables 1 and 2. The mean disease duration ranged from 2.0 to 19 years.

### Biochemical parameters

#### Serum calcium

**Fig. 2:**
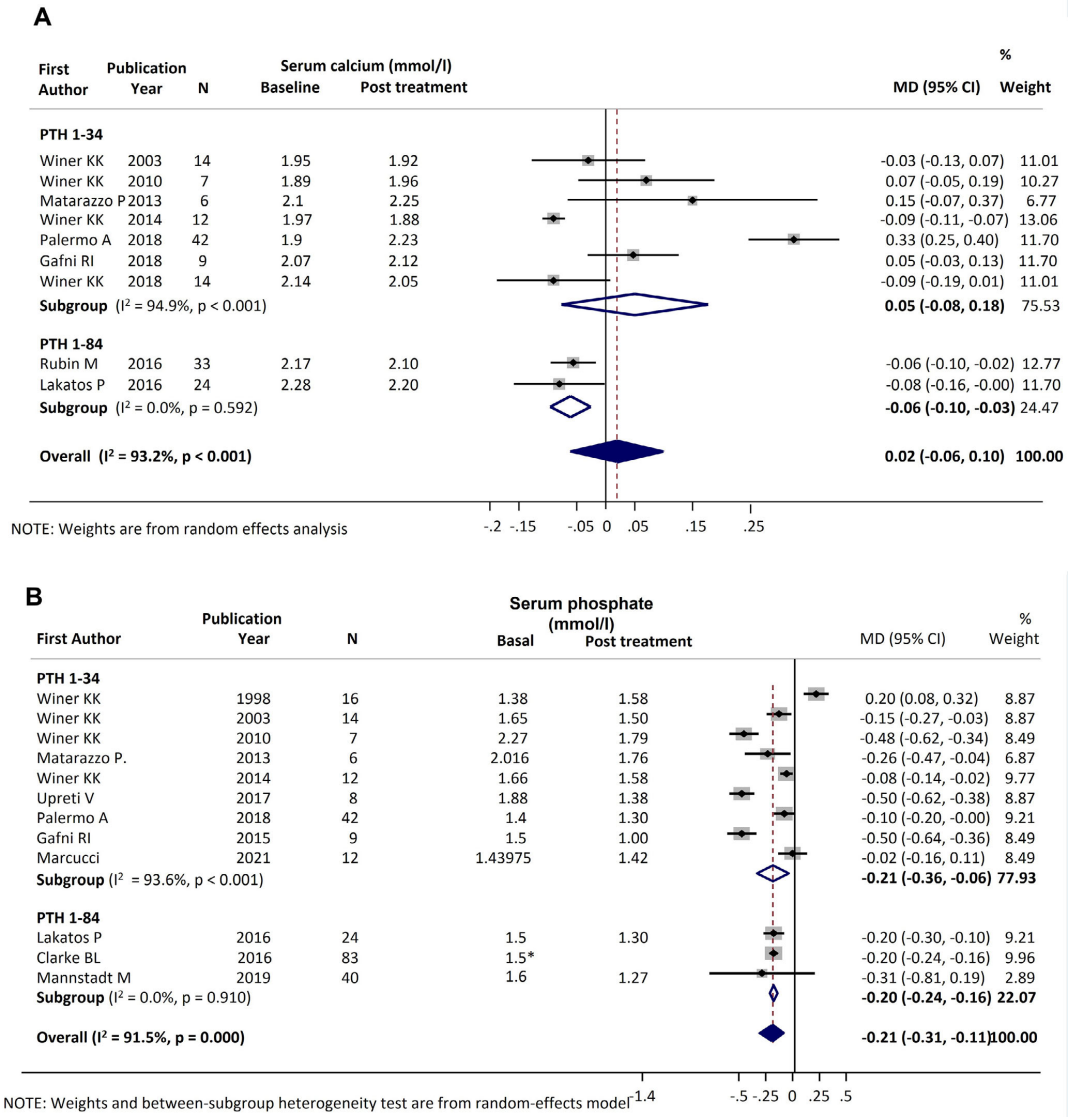
Forest plot of serum calcium (*A*) and phosphate (*B*) levels during PTH_1−34_ and PTH_1−84_ therapy. *Clarke and colleagues^(^ ^39^ ^)^ provide only change.

Meta‐analysis of serum calcium change from baseline was performed with data from nine studies (161 patients): seven on PTH_1−34_^(^ ^20^, ^26^, ^31^, ^32^, ^33^, ^34^, ^35^ ^)^ and two on PTH_1−84_.^(^ ^36^, ^37^ ^)^ Funnel plot for distribution of studies on changes in serum calcium is available in Supplementary Figure S1. The treatment had no significant effect on calcium levels (MD = 0.02 mmol/L; 95% CI, −0.06 to 0.10 mmol/L; *p* = 0.636) (*I* ^2^ = 93.2%; *p* < 0.001) (Fig. 2*A*) and there was no difference in the overall effect size between the two treatment groups (test: Q_b_ = 3.26, *p* = 0.071). Analysis limited to RCTs also revealed a nonsignificant change (MD = 0.014; 95% CI, −0.083 to 0.11; *p* = 0.770),^(^ ^31^, ^32^ ^)^ with no significant heterogeneity (*I* ^2^ = 39%, *p* = 0.200).

Subgroup analysis based on calcium supplementation (discontinuation at baseline^(^ ^20^, ^26^, ^32^, ^33^ ^)^ or down‐titration during the study,^(^ ^31^, ^34^, ^35^, ^36^, ^37^ ^)^) revealed no major differences (*p* = 0.13). The analysis of controls showed a nonsignificant change in calcium levels,^(^ ^31^, ^32^ ^)^ with no heterogeneity. The difference in calcium level variation between patients and controls was nonsignificant (MD = −0.01; 95% CI, −0.11 to 0.09; *p* = 0.833). Analysis of albumin‐corrected calcium is reported online in Appendix S1. Overall, the evidence converges toward a neutral effect size of PTH therapy on serum calcium.

#### Serum phosphate

Data on serum phosphate levels were extracted from 12 studies (254 patients): nine on PTH_1−34_^(^ ^19^, ^23^, ^26^, ^31^, ^32^, ^33^, ^34^, ^35^, ^38^ ^)^ and three on PTH_1−84_.^(^ ^37^, ^39^, ^40^ ^)^ Funnel plot for distribution of studies on changes in serum calcium is available in Supplementary Figure S2. The treatments significantly reduced phosphate levels, with an overall pooled MD of −0.21 mmol/L (95% CI, −0.31 to −0.11 mmol/L; *p* < 0.001) (*I* ^2^ = 91.5%; *p* < 0.001) (Fig. 2*B*). There was no difference between the efficacy of the two PTH treatments (Q_b_ = 0.01, *p* = 0.936). Subgroup analysis on RCTs confirmed a significant reduction in serum phosphate (MD = −0.26; 95% CI, −0.43 to −0.10; *p* = 0.001; *I* ^2^ = 87.6%; *p* < 0.001),^(^ ^31^, ^32^, ^39^ ^)^ but not in the control groups^(^ ^10^, ^32^, ^39^ ^)^ (MD = −0.13; 95% CI, −0.26 to −0.004 mmol/L; *p* = 0.044). Ultimately, the analyses revealed that PTH therapies have a large size effect in lowering serum phosphate levels.

#### Calcium phosphate product

**Fig. 3:**
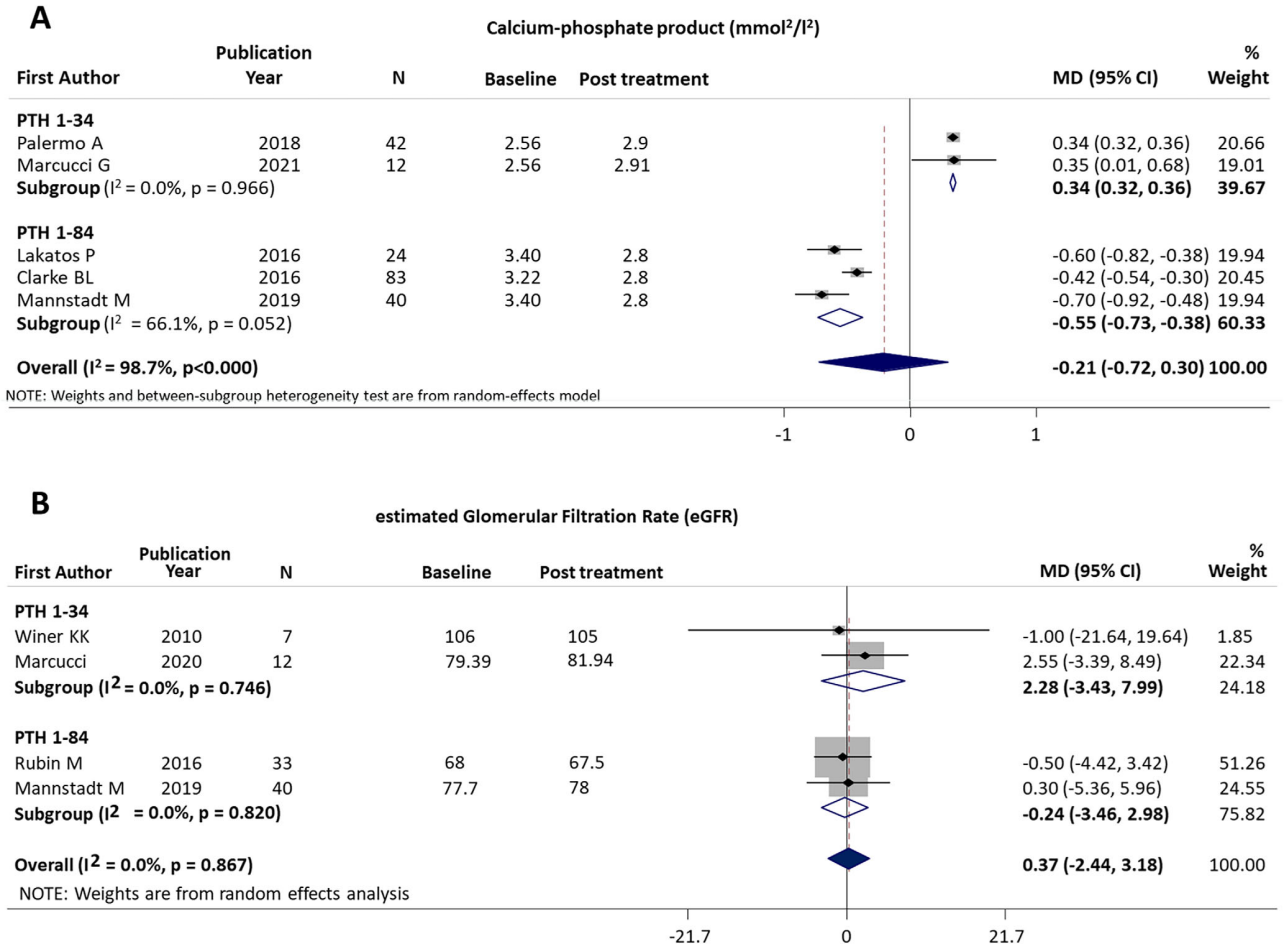
Forest plot of change in serum calcium‐phosphate product (*A*) and eGFR (*B*).

Elevated calcium phosphate products has been considered a marker of increased cardiorenal risk.^(^ ^41^ ^)^ Two studies using PTH_1−34_^(^ ^19^, ^35^ ^)^ (54 patients) reported data on calcium phosphate product, showing a small but statistically significant increase (MD = 0.34 mmol^2^/L^2^; 95% CI, 0.32 to 0.36; *p* < 0.001; *I* ^2^ = 0%; *p* = 0.966). In contrast, extraction of data from the three studies^(^ ^37^, ^39^, ^40^ ^)^ (147 patients) using PTH_1−84_ revealed a significant reduction (MD = −0.55 mmol^2^/L^2^; 95% CI, −0.73 to −0.38; *p* < 0.001; *I* ^2^ = 66.1%; *p* = 0.052; Fig. 3*A*). A significant difference between the two PTH treatments was confirmed in the overall effect size (Q_b_ = 100.45, *p* < 0.001), with only PTH_1−84_ able to reduce calcium phosphate product. However, in the two studies investigating PTH_1−34_, a marginal increase in calcium^(^ ^35^ ^)^ and albumin‐corrected calcium^(^ ^19^ ^)^ was observed.

#### Estimated glomerular filtration rate

Results on the effects of PTH therapy on estimated glomerular filtration rate (eGFR) were extracted from four studies, two on PTH_1−34_ (24 patients)^(^ ^19^, ^31^ ^)^ and two on PTH_1−84_ (82 patients).^(^ ^36^, ^40^ ^)^ Methods of eGFR analysis varied between the studies. The results of the meta‐analysis showed an overall pooled nonsignificant change (MD = 0.37 mL/min; 95% CI, −2.44 to 3.18; *p* = 0.797; Fig. 3*B*).

Treatment subgroup analysis for eGFR revealed an MD of 2.28 mL/min (95% CI, −3.43 to 7.99; *p* = 0.434) after PTH_1−34_ and −0.24 mL/min (95% CI, −3.46 to 2.98; *p* = 0.884) after PTH_1−84_. The overall heterogeneity was not significant (*I* ^2^ = 0%; *p* = 0.867). The analyses showed no reduction in eGFR during treatment.

#### Serum 25(OH)D and 1,25 dihydroxivitamin D

**Fig. 4:**
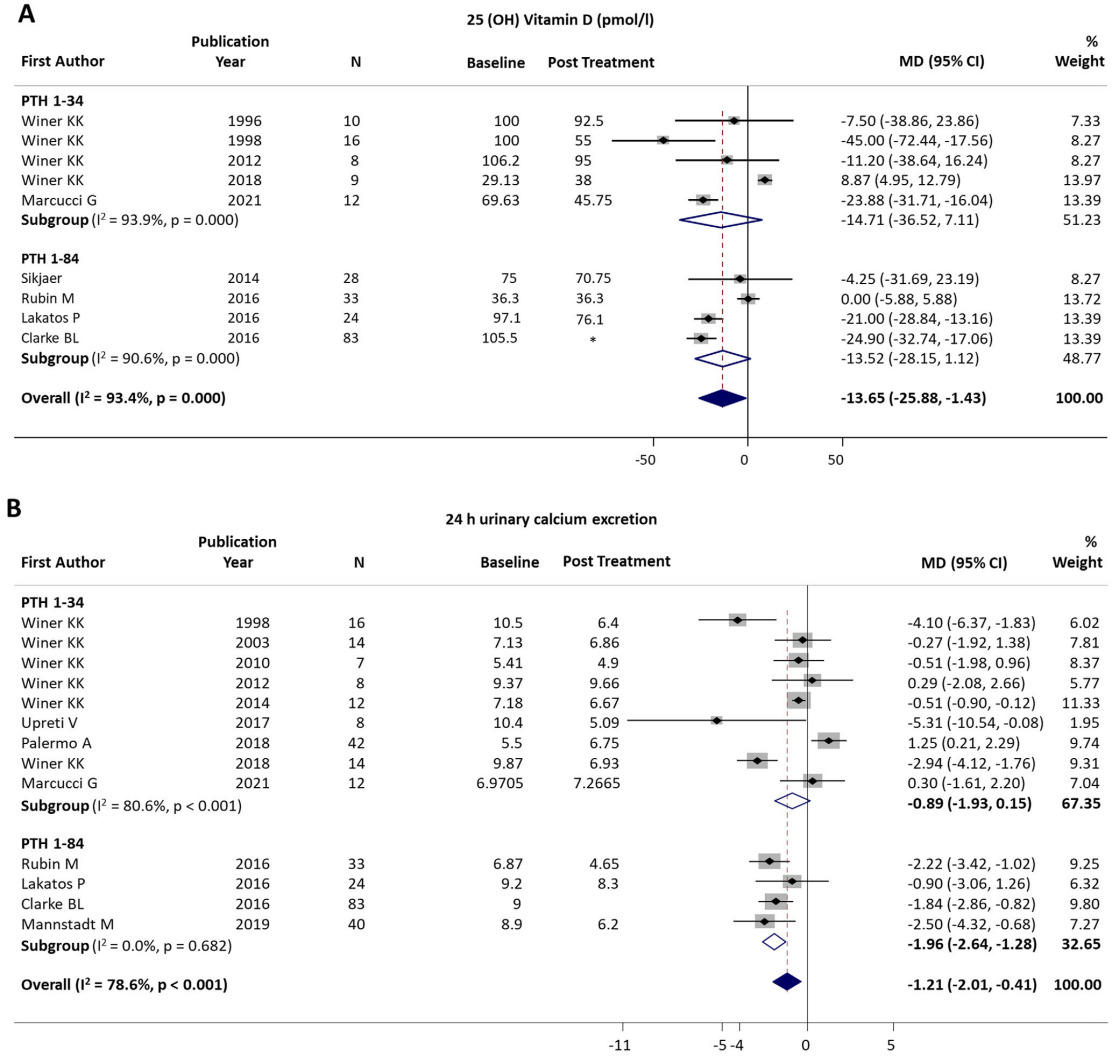
Forest plot of change in 25(OH) vitamin D (*A*) and 24‐hour urinary calcium excretion (*B*). *Clarke and colleagues^(^ ^39^ ^)^ reported variation in 25(OH) vitamin D only.

Effects on 25(OH) vitamin D levels were reported in nine studies (223 patients): five on PTH_1−34_^(^ ^10^, ^19^, ^20^, ^23^, ^25^ ^)^ and four on PTH_1−84_.^(^ ^36^, ^37^, ^39^, ^42^ ^)^ A significant overall pooled reduction was observed (MD = −13.65 pmol/L; 95% CI, −25.88 to 1.43 pmol/L; *p* = 0.029). The heterogeneity was high (*I* ^2^ = 93.4%; *p* < 0.001) (Fig. 4*A*). No difference was found between the two PTH treatments (Q_b_ = 0.01, *p* = 0.929). Effects on 1,25 dihydroxivitamin D (1,25(OH)_2_ vitamin D) levels were reported in five studies (171 patients): four on PTH_1−34_^(^ ^10^, ^19^, ^23^, ^25^ ^)^ and one on PTH_1−84_.^(^ ^39^ ^)^ A nonsignificant overall increase was observed (MD = 3.03 pmol/L; 95% CI, −8.94 to 14.99 pmol/L; *p* = 0.620). Taken as a whole, the results suggest that there is a treatment‐related drop in 25(OH)D, even if data on the cholecalciferol supplementation was unavailable in most studies, and an effect of its reduction cannot be excluded.

#### Urinary calcium excretion

The effects of PTH therapy on urinary calcium excretion, measured on 24‐hour urine collection, were reported in 13 studies (313 patients): nine on PTH_1−34_^(^ ^19^, ^20^, ^23^, ^25^, ^26^, ^31^, ^32^, ^35^, ^38^ ^)^ and four on PTH_1−84_.^(^ ^36^, ^37^, ^39^, ^40^ ^)^ Funnel plot for distribution of studies on changes in 24‐hour urinary calcium excretion is available in Supplementary Figure S3. The meta‐analysis revealed an overall reduction of −1.21 mmol/24 h (95% CI, −2.03 to −0.41 mmol/24 h; *p* = 0.003). Heterogeneity was high (*I* ^2^ = 78.6%, *p* < 0.001) (Fig. 4*B*). No difference was found between the two PTH treatments (Q_b_ = 2.85, *p* = 0.091).

Subgroup analysis of randomized trials confirmed a significant reduction in urinary calcium excretion (MD = −1.05; 95% CI, −1.95 to −0.14; *p* = 0.023),^(^ ^23^, ^25^, ^26^, ^31^, ^32^, ^39^ ^)^ with moderate heterogeneity (*I* ^2^ = 67.3%, *p* = 0.009). Subgroup analysis based on calcium supplementation (discontinuation at baseline^(^ ^20^, ^23^, ^25^, ^26^, ^32^ ^)^ versus titration during study^(^ ^31^, ^35^, ^36^, ^37^ ^)^) showed no difference (*p* = 0.434). There was no reduction in the pooled MD in controls taking conventional treatment (MD = −0.072 mmol/24 h; 95% CI, −2.41 to 0.97 mmol/24 h; *p* = 0.403) (*I* ^2^ = 82.1%; *p* = 0.004). Overall, the evidence indicates a consistent improvement in urinary calcium excretion achieved with PTH therapy, regardless of the calcium supplementation regimen.

#### Urinary phosphate excretion

The effects on urinary phosphate excretion, measured on 24‐hour urine collection, could be extracted from three studies using PTH_1−34_^(^ ^19^, ^23^, ^31^ ^)^ (41 patients), with high heterogeneity (*I* ^2^ = 82.5%, *p* = 0.003). The change was nonsignificant (MD = 0.66; 95% CI, −6.82 to 8.14; *p* = 0.862).

### Bone turnover markers and bone mineral density

Treatment data on osteocalcin were only available in three studies (50 patients): two on PTH_1−34_^(^ ^23^, ^34^ ^)^ and one on PTH_1−84_.^(^ ^37^ ^)^ Overall, there was an increase in osteocalcin under PTH treatment (MD = 28.3 μg/L; 95% CI, 3.69 to 52.83; *p* = 0.024), with a high heterogeneity (*I* ^2^ = 91.6%, *p* < 0.001).

Treatment data on alkaline phosphatase (ALP) were available in five studies on PTH_1−34_^(^ ^20^, ^23^, ^33^, ^35^, ^38^ ^)^ (87 patients). The meta‐analysis showed a pooled significant increase in ALP (MD = 58.79 U/L; 95% CI, 34.31 to 83.27 U/L; *p* < 0.001), with moderate heterogeneity (*I* ^2^ = 75.3%, *p* = 0.003), mainly due to the size of difference rather than direction of change between studies. Similar findings were observed for bone alkaline phosphatase (BAP) after PTH_1−34_^(^ ^19^, ^34^ ^)^ and PTH_1−84_^(^ ^37^, ^40^ ^)^ (94 patients), with a significant increase in BAP (MD = 17.8 μg/L; 95% CI, 8.65 to 27.02; *p* < 0.001). Two studies on PTH_1−84_ (73 patients) reported an increase in c‐terminal telopeptide (CTX)^(^ ^37^, ^40^ ^)^ during treatment (MD = 445.90 μg/mL; 95% CI, 53.74 to 838.07; *p* = 0.026), but with high heterogeneity (*I* ^2^ = 93.7%; *p* < 0.001).

The meta‐analysis of changes in lumbar spine, neck and total femoral bone mineral density (BMD) was conducted on two studies on PTH_1−84_^(^ ^36^, ^37^ ^)^ and one on PTH_1−34_^(^ ^38^ ^)^ (65 patients). The treatment induced a significant increase in lumbar spine BMD (MD = 0.045 g/cm^2^; 95% CI, 0.015 to 0.074 g/cm^2^; *p* = 0.003), with no heterogeneity (*I* ^2^ = 0%; *p* = 0.810). No significant change was observed for total hip, femoral neck, or distal radius BMD (see the Supplementary Results in Appendix S1).

Overall, despite the heterogeneity between studies, which could affect the results, PTH therapies increased ALP, BAP, osteocalcin, and CTX, accompanied by an increase in lumbar spine BMD.

### Conventional therapy

#### Calcium supplementation

**Fig. 5:**
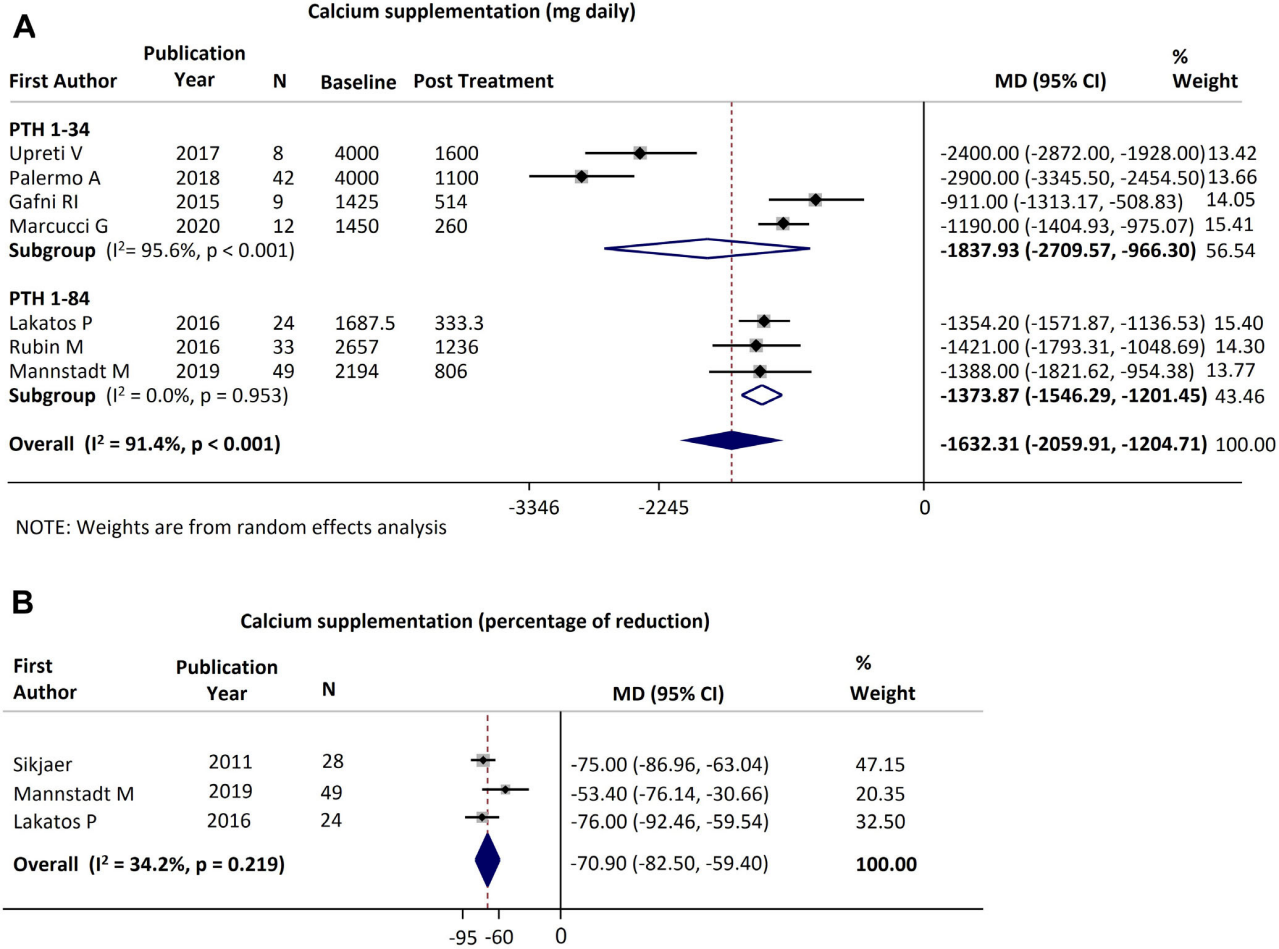
Forest plot of change in calcium supplementation, of study which provided data expressed as g/daily (*A*) and percentage of reduction (*B*).

Seven studies reported data on calcium supplementation needs (177 patients): four on PTH_1−34_^(^ ^19^, ^34^, ^35^, ^38^ ^)^ and three on PTH_1−84_.^(^ ^36^, ^37^, ^40^ ^)^ Pooled MD revealed a reduction in calcium dosage (−1.63 g/daily; 95% CI, −2.06 to −1.20 g; *p* < 0.001) (*I* ^2^ = 91.4%; *p* < 0.001) (Fig. 5*A*), with similar results for both PTH treatments (Q_b_ = 0.93; *p* = 0.335).

Three PTH_1−84_ studies^(^ ^37^, ^40^, ^43^ ^)^ (101 patients) provided SE for the percentage of reduction and were included in the meta‐analysis. The pooled reduction was −70.9% (95% CI, −82.5% to −59.4%; *p* < 0.001; *I* ^2^ = 34.2%; Fig. 5*B*). Fourteen studies^(^ ^10^, ^11^, ^19^, ^26^, ^32^, ^33^, ^34^, ^35^, ^36^, ^37^, ^38^, ^40^, ^43^, ^44^ ^)^ reported the mean variation in percentage of daily calcium supplementation, and the extracted median of variation was −73% (range, −100% to −9%; Supplementary Fig. S4).

Complete withdrawal from calcium supplementation was reported in seven studies.^(^ ^10^, ^19^, ^26^, ^32^, ^37^, ^40^, ^43^ ^)^ The estimated rate of discontinuation was 46.7% in patients receiving PTH_1−84_ (49/105) and 57% in those receiving PTH_1−34_ (20/35) (additional data are reported in Appendix S1).

Overall, most patients receiving PTH were able to discontinue or substantially reduce (>1 g/day) their calcium supplementation.

#### Active vitamin D supplementation

**Fig. 6:**
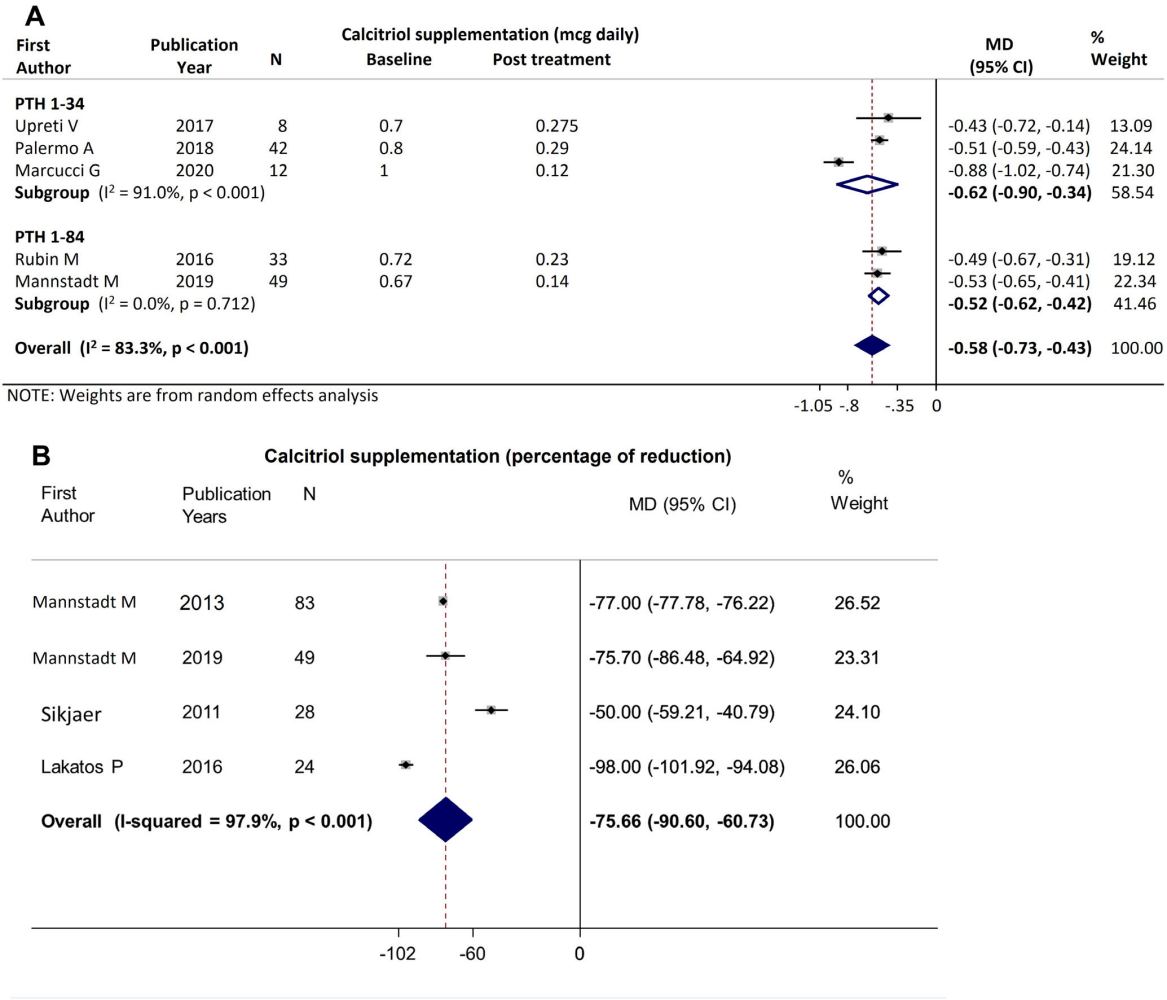
Forest plot of change in calcitriol supplementation, of study which provided data expressed as g/daily (*A*) and percentage of reduction (*B*).

Five studies reported data on active vitamin D supplementation (144 patients): three studies on PTH_1−34_^(^ ^19^, ^35^, ^38^ ^)^ and two on PTH_1−84_.^(^ ^36^, ^40^ ^)^ Pooled MD showed a 0.58 μg reduction in the daily dosage (95% CI, −0.73 to −0.43 μg; *p* < 0.001) associated with PTH therapy, but with significant heterogeneity (*I* ^2^ = 83.3%; *p* < 0.001; Fig. 6*A*). No difference in effect size was found between PTH_1−34_ and PTH_1−84_ (Q_b_ = 0.47; *p* = 0.491). In the four PTH_1−84_ studies that provided data as percentage and were suitable for the analysis (184 patients),^(^ ^11^, ^37^, ^40^, ^43^ ^)^ the pooled estimated MD was −75.7% (95% CI, −90.6% to −60.7; *p* < 0.001), with high heterogeneity (*I* ^2^ = 97.9%; *p* < 0.001), as reported in Fig. 6*B*.

A sensitivity analysis on RCTs showed a significant reduction in calcitriol supplementation (MD = −63.9%; 95% CI, −90.35 to −37.46%; *p* < 0.001),^(^ ^11^, ^43^ ^)^ with high heterogeneity (*I* ^2^ = 96.9%; *p* < 0.001).

Thirteen studies^(^ ^11^, ^19^, ^23^, ^26^, ^32^, ^34^, ^35^, ^36^, ^37^, ^38^, ^40^, ^43^, ^44^ ^)^ also reported the reduction in calcitriol supplementation as a percentage (median of variation −83%; range, −100% to −23%) (Supplementary Fig. S5). Complete withdrawal from calcitriol replacement therapy was reported in PTH_1−34_^(^ ^19^, ^23^, ^26^, ^32^, ^34^ ^)^ (96.2%; 95% CI, 89.3% to 99.2%) as well as PTH_1−84_^(^ ^36^, ^37^, ^40^, ^43^ ^)^ studies (65.7%; 95% CI, 56.98% to 73.65%). Data for treatment versus controls are reported in Appendix S1.

### QoL

Studies evaluating QoL in patients receiving PTH therapies used various versions of the short‐form health survey (SF‐36) questionnaire. To obtain pooled results, SMD was calculated when possible. Overall, one study on PTH_1−34_^(^ ^45^ ^)^ and two studies on PTH_1−84_^(^ ^46^, ^47^ ^)^ (194 patients) were included in the meta‐analysis. This revealed a significant PTH‐related improvement in both the PCS and the MCS, with an SMD of 2.21 (95% CI, 0.32 to 4.11; *p* = 0.022) and 2.30 (95% CI, 0.33 to 4.26; *p* = 0.022), respectively. Heterogeneity was very high (*I* ^2^ = 98%; *p* < 0.001), mainly due to the difference in the magnitude of effect, rather than the direction of change.

### Adverse events

Data on adverse events were described differently across studies. Data were available in a total of 18 studies (354 patients): 12 on PTH_1−34_^(^ ^10^, ^19^, ^20^, ^23^, ^25^, ^26^, ^31^, ^33^, ^34^, ^35^, ^38^, ^45^, ^48^ ^)^ and six on PTH_1−84_.^(^ ^11^, ^36^, ^37^, ^40^, ^43^, ^44^ ^)^ The median study duration was 21 months (range, 3.5 to 60 months) for PTH_1−34_ and 18 months for PTH_1−84_ (range, 2 to 72).

Overall, 236 patients experienced some adverse events during PTH therapy (estimated percentage: 66.7%; 95% CI, 61.5% to 71.6%). The majority of events were mild and not correlated with PTH treatment. Twenty‐four of the 117 patients treated with PTH_1−34_ had an adverse event^(^ ^10^, ^25^, ^26^, ^33^, ^38^, ^45^, ^49^ ^)^ (estimated percentage 20.5%; 95% CI, 0.14% to 29%), compared with 212 of the 237 patients in the PTH_1−84_ studies (estimated percentage 89.5%; 95% CI, 84.8% to 93.1%).^(^ ^11^, ^37^, ^40^, ^43^, ^44^ ^)^

Of the six studies^(^ ^11^, ^26^, ^33^, ^37^, ^43^, ^44^ ^)^ providing the number of adverse events and the number of patients experiencing at least one event, the two studies on PTH_1−34_ had an average of zero events per patient, whereas the four studies on PTH_1−84_ had an average of seven events per patient.

The most frequently reported events were non–treatment‐related infectious diseases, headache, paresthesia, gastrointestinal symptoms, kidney disease, and hypercalciuria. The few adverse events considered treatment‐related by study investigators were hypercalcemia, nausea, constipation, headache, injection site reactions, and bone pain. No evidence of osteosarcoma or other malignancies was reported in patients treated with PTH. A full description of adverse events is available in Appendix S1 and in Supplementary Table S1.

#### Serious adverse events

Serious adverse events (SAEs) were reported in 11 studies (330 patients). Overall, 30 patients reported at least one SEA (estimated percentage 9.1%; 95% CI, 6.2% to 12.7%). The estimated percentage of patients experiencing a SAE throughout the study observation period was 2.2% (95% CI, 0.3% to 7.6%) for PTH_1−34_^(^ ^23^, ^25^, ^26^, ^33^, ^35^, ^38^ ^)^ and 11.8% (95% CI, 8.0% to 16.7%) for PTH_1−84_.^(^ ^11^, ^37^, ^40^, ^43^, ^44^ ^)^ This percentage does not take into account the results from Rubin and colleagues,^(^ ^36^ ^)^ who described nine SAEs without reporting the number of patients. The large majority of SAEs were not considered PTH‐related.

#### Treatment‐related adverse events

Pooled data for the two studies^(^ ^11^, ^43^ ^)^ that also reported adverse events in controls showed a nonsignificant association with treatment, with a pooled relative risk (RR) of 0.97 (95% CI, 0.91 to 1.04; *p* = 0.932), and nonsignificant heterogeneity between the studies (*I* ^2^ = 47.5%; *p* = 0.167). Both these studies showed an increased risk of SAEs in patients receiving PTH therapy compared to controls, but results on pooled risk were nonsignificant (RR = 1.35; 95% CI, 0.58 to 3.16; *p* = 0.491).

Data on hypocalcaemia were available for patients and controls. There was a reduced relative risk in patients taking PTH therapy in one study^(^ ^43^ ^)^ and an increased—albeit nonsignificant—risk in the other.^(^ ^11^ ^)^ However, pooled data confirmed a nonsignificant difference in relative risk for hypocalcaemia between patients taking PTH therapy and controls (RR = 0.74; 95% CI, 0.22 to 2.51; *p* = 0.626); the heterogeneity between the studies was high (*I* ^2^ = 82%; *p* = 0.018). Similarly, pooled data showed a nonsignificant difference in relative risk for hypercalcemia between patients taking PTH therapy and controls (RR = 3.98; 95% CI, 0.77 to 20.55; *p* = 0.099; *I* ^2^ = 56.5%; *p* = 0.130).

## Discussion

Through a systematic analysis of all published clinical trials, we collated the largest group to date of patients treated with PTH‐receptor agonists and demonstrated their efficacy in restoring serum phosphate levels, reducing calcium excretion and reactivating bone turnover—all issues that are not addressed by any alternative treatment. These therapies also enabled the reduction or discontinuation of oral calcium and vitamin D supplementation, and had a relatively good safety profile.

The first clinical study on PTH replacement therapy in hypoparathyroidism dates back to 1996.^(^ ^10^ ^)^ Despite the 25 years elapsed since then, very few randomized trials have been conducted to date, and none have compared PTH_1−34_ and PTH_1−84_. Given the rarity of this disease, the few trials conducted were small in size and heterogeneous in outcomes or design, limiting the ability to draw robust conclusions. PTH replacement therapy is not currently used as standard clinical practice according to the European guidelines.^(^ ^4^ ^)^ However, although the conventional therapies are generally able to control hypocalcaemia, they leave several clinical issues unaddressed: hyperphosphatemia, hypercalciuria, and nephrocalcinosis are a significant burden for many patients, for whom PTH treatment might well be a better alternative.

One of the concerns in the treatment of hypoparathyroidism with calcium and calcitriol supplements is the delicate balance between overtreatment and symptomatic hypocalcaemia. Our meta‐analysis showed PTH replacement therapy could be considered neither superior nor inferior to oral supplements in controlling serum calcium levels. This may be because the patients enrolled in clinical trials were mostly on target for calcium at the baseline, and their oral calcium supplements were significantly reduced or discontinued during studies. It is worth noticing that studies were heterogenous in duration. We showed that PTH therapies are safe with respect to the risk of hypocalcemic or hypercalcemic episodes.

An unmet need in hypoparathyroidism is combating the associated rise in serum phosphate levels, which are largely unaffected by conventional therapies.^(^ ^12^, ^50^ ^)^ Active vitamin D is unable to match the phosphaturic action of PTH and can even worsen hyperphosphatemia, by promoting intestinal phosphate absorption. Our meta‐analysis revealed a large posttreatment reduction in serum phosphate and a significant difference between patients receiving PTH therapies and controls. PTH_1−34_ and PTH_1−84_ showed a similar efficacy in lowering serum phosphate.

Increased serum phosphate is considered a predictor of poor health^(^ ^51^, ^52^, ^53^ ^)^ and has been correlated with various complications of hypoparathyroidism, including an increased risk of infectious diseases.^(^ ^54^ ^)^ Neurological and neuropsychiatric complications^(^ ^55^ ^)^ are also common, including basal ganglia calcifications, which seem to be correlated with calcium phosphate product.^(^ ^56^ ^)^ Surprisingly, we found that PTH_1−84_ was more effective than PTH_1−34_ in reducing calcium phosphate product. This finding, which needs further confirmation as only four studies could be included in the analysis, suggests that PTH_1−84_ is more effective in preventing ectopic calcifications.

Another challenge in managing hypoparathyroidism is the control of urinary calcium excretion. Conventional therapy with active vitamin D does not restore natural PTH‐mediated tubular calcium reabsorption.^(^ ^57^ ^)^ Other strategies, such as the use of thiazide diuretics, are not always feasible or tolerated.^(^ ^58^ ^)^ Our meta‐analysis found that PTH replacement therapy is not only safe but is also decidedly superior to conventional therapy in controlling urinary calcium excretion.

The reduced urinary calcium achieved through PTH replacement therapy lowers the risk of kidney stones, which could otherwise contribute to a progressive deterioration of renal function, and ultimately end‐stage kidney disease.^(^ ^7^ ^)^ Furthermore, although the sample size was not powered to ultimately address the effects on eGFR, no treatment‐associated reduction at up to 6 years of follow‐up.^(^ ^36^ ^)^ This is an important finding, given that conventional therapy for hypoparathyroidism is associated with a progressive decline in renal function.^(^ ^55^ ^)^ In short, the analysis provides convincing evidence that PTH replacement therapy is superior to any available treatment at restoring a normal electrolyte balance in relation to calcium and phosphate reabsorption and excretion.

Low bone turnover is a major clinical concern in hypoparathyroid patients. BMD data revealed only a marginal increase in lumbar spine density after PTH replacement therapy, with no effects on total hip, femoral neck, or distal radius. However, data were only available for 92 patients, with variable treatment duration (6 and 36 months). In any case, BMD may be a misleading marker for bone health in hypoparathyroidism: despite a normal or even increased BMD compared to age and sex‐matched controls,^(^ ^59^ ^)^ there were conflicting data on the risk of fractures. Some studies reported that patients with hypoparathyroidism have an increased risk of fractures^(^ ^54^, ^60^ ^)^ whereas others reported no increase in the risk of fracture^(^ ^8^ ^)^ or a reduced risk of upper extremity fracture in postsurgical hypoparathyroidism.^(^ ^6^ ^)^ In contrast with the results for BMD, both bone formation and resorption markers significantly increased during PTH replacement therapy.

Calcium supplements are often poorly tolerated or absorbed, leading to dose fractioning and the inconvenience of multiple daily administrations. Meta‐analysis of the published data confirmed that PTH replacement is effective in reducing calcium and calcitriol therapy. This reduction also appears to be clinically meaningful, with a mean reduction in calcium supplementation of more than 70% of the daily calcium dosage (~1.5 g/daily) or calcitriol (or equivalent) replacement (~0.60 μg/daily). Withdrawal of oral supplementation was also possible in a large percentage of patients after transitioning to PTH replacement therapy. Interestingly, no difference was found between the PTH formulations, suggesting that both are equally effective in this respect. PTH replacement therapy could thus be a valid alternative to calcium supplements in terms of adherence and overall perceived wellness.

Patients with hypoparathyroidism generally report decreased QoL. This is in part due to so‐called “brain fog,” which persists even when serum calcium levels are well‐controlled.^(^ ^61^ ^)^ Our meta‐analysis revealed a significant improvement in both the mental and physical composite score of the SF‐36 questionnaire after PTH replacement therapy, although results were only available from uncontrolled studies. Long‐term studies^(^ ^21^ ^)^ not included in this meta‐analysis show similar findings.

PTH replacement therapy itself is not free from side effects. Although the data on its safety are reassuring, a significant number of adverse events have been reported, especially in the most recent trials with PTH_1−84_. However, most of these should be ascribed to the fragility of hypoparathyroid patients, rather than the treatments themselves. Our meta‐analysis did not show any increased risk of total adverse events or serious adverse events compared to controls in patients receiving PTH_1−34_ and PTH_1−84_.

In the past, several concerns were raised after the publication of animal studies on PTH_1−34_ therapy for osteoporosis that described an increased risk of malignancies,^(^ ^62^ ^)^ leading to an initial restriction of the permitted treatment duration in many countries. However, for patients with hypoparathyroidism, PTH therapy should be considered as the replacement of a missing hormone and is thus not comparable to the use of PTH_1−34_ to treat osteoporosis. As further support, long term studies in hypoparathyroidism patients did not report malignancies in the PTH group.^(^ ^21^, ^48^ ^)^

Published data seem to highlight a higher number of adverse events with PTH_1−84_ than with PTH_1−34_. However, this may be affected by other aspects, such as study design and differences in how side effects were collected. For example, the REPLACE study (A Randomized, Double‐Blind, Placebo‐Controlled, Phase 3 Study to Investigate the Use of NPSP558, a Recombinant Human Parathyroid Hormone (rhPTH[1‐84]) for the Treatment of Adults With Hypoparathyroidism)^(^ ^11^, ^39^ ^)^ reported a high number of mild adverse events, which might have increased the overall rate of side effects of PTH_1−84_ compared to PTH_1−34_. It is also important to consider that only two trials (one for each treatment) provided data for both patients and controls, and both the incidence and the number of adverse events were similar across both these groups. Further head‐to‐head comparative studies of PTH_1−84_ and PTH_1−34_ could confirm or reject these hypotheses.

There are several limitations affecting the results of this meta‐analysis, mostly in relation to the small number and the heterogeneity of the studies. However, hypoparathyroidism is a rare disease, and a relatively small sample size is to be expected. Several trials provided results from the same cohort in different reports. Because this has to be avoided in a rigorous meta‐analysis, we also reduced the number of eligible studies included. We made a significant effort to identify overlapping cohorts, enabling us to focus on high‐quality data. Unfortunately, over the years several patients have participated in different clinical trials and cannot be considered naïve to PTH treatment on enrollment in a given study. Moreover, not all studies reported similar outcomes or full baseline and posttreatment values, further narrowing the available evidence. There are also several important differences in titration regimens, dosing, and inclusion criteria that contribute to the heterogeneity of the results, possibly masking differences and reducing their generalizability. Furthermore, the data could not be analyzed in relation to different etiologies of hypoparathyroidism, which could be a confounding factor. A recent study by Winer and colleagues^(^ ^63^ ^)^ found that patients with genetic mutations required higher doses of PTH_1−34_ than did patients with postsurgical or idiopathic hypoparathyroidism. Finally, most of the studies were sponsored, with only 10 nonprofit studies included.

Although the analysis of the overall effects of PTH replacement therapy is very convincing, the comparison between PTH_1−34_ and PTH_1−84_ highlights the need for further studies, possibly including newer formulations such as TransCon PTH, an investigational long‐acting prodrug of PTH. The efficacy of this long‐acting prodrug of PTH_1−34_ in reducing conventional treatment, achieving biochemical control and improving QoL was recently demonstrated in a phase 2 trial.^(^ ^64^ ^)^

Future studies should ideally address specific aspects, such as more standardized analyses of the effects of PTH replacement therapy on QoL and the risk of bone adverse events in hypoparathyroidism. There is also a need to evaluate its effects on other possible complications of hypoparathyroidism, such as basal ganglia calcifications,^(^ ^65^ ^)^ alterations in immune profile,^(^ ^2^, ^3^ ^)^ nephrocalcinosis and kidney stone formation,^(^ ^66^ ^)^ and cardiovascular complications.^(^ ^67^, ^68^ ^)^ Future trials should include patients who do not achieve adequate control with conventional therapies, because most subjects included in this meta‐analysis were at target for serum calcium on enrollment in their respective studies. Finally, because the use of PTH_1−34_ has been advocated for hypoparathyroidism in many countries, a head‐to‐head comparison between full PTH_1−84_ and its fragment PTH_1−34_ will bring to light any differences that could not be detected from the available data.

## Conclusions

Our meta‐analysis of the largest available number of treated patients demonstrates that PTH replacement therapy is well‐tolerated, safe, and effective in the management of hypoparathyroidism. Unlike conventional supplementation, it has beneficial effects on serum phosphate, calcium‐phosphate product, and urinary calcium excretion, which are currently the unmet needs in chronic hypoparathyroidism. Basing on available data, no significant differences between PTH_1−34_ and PTH_1−84_ have been demonstrated in most outcomes, except for a beneficial effect of PTH_1−84_ only in reducing calcium‐phosphate product. It also improves several extrarenal outcomes, such as QoL and bone turnover. In addition to its current indication as second‐line treatment in patients with refractory hypocalcaemia, our results support the use of PTH replacement therapy in patients in whom hyperphosphatemia and increased urinary calcium excretion are a clinical concern.

## Author Contributions

**Giulia Puliani:** Data curation; investigation; resources; writing – original draft. **Valeria Hasenmajer:** Data curation; investigation; resources; writing – original draft. **Ilaria Simonelli:** Formal analysis; methodology; validation; visualization. **Valentina Sada:** Data curation; investigation; resources. **Riccardo Pofi:** Validation. **Marianna Minnetti:** Validation. **Alessia Cozzolino:** Validation. **Nicola Napoli:** Supervision; writing – review and editing. **Patrizio Pasqualetti:** Methodology; supervision. **Daniele Gianfrilli:** Supervision; writing – review and editing. **Andrea M. Isidori:** Conceptualization; methodology; project administration; supervision; writing – review and editing.

## Conflicts of Interest

AMI does consultancy for Novartis, Takeda, Recordati and Sandoz companies and has received research grants and honorariums from Shire, IPSEN and Pfizer company; NN does consultancy for Takeda and Lilly companies; no other relationships or activities that could appear to have influenced the submitted work.

## Supporting information


**Appendix S1** Supplementary Information

**Supplementary Fig. S1** Funnel plot for distribution of studies on changes in serum calcium in patients treated by PTH 1‐34 and controls.

**Supplementary Fig. S2** Funnel plot for distribution of studies on changes in serum phosphate levels in patients treated by PTH 1‐34 and controls.

**Supplementary Fig. S3** Funnel plot for distribution of studies on changes in 24‐hour urinary calcium excretion levels in patients treated by PTH 1‐34 and controls.

**Supplementary Fig. S4** Bubble chart of percentage of reduction in calcium supplementation after PTH 1‐34 (dark gray) and PTH 1‐84 (light gray) treatment. Trials in which discontinuation of conventional therapy was not titrated according to serum calcium are striped.

**Supplementary Fig. S5** Bubble chart of percentage of reduction in calcitriol supplementation after PTH 1‐34 (dark gray) and PTH 1‐84 (light gray) treatment. Trials in which discontinuation of conventional therapy was not titrated according to serum calcium are striped.

## Data Availability

Data sharing is not applicable to this article as no new data were created in this study.
