# Supplementary material for: Safety and Efficacy of PTH 1‐34 and 1‐84 Therapy in Chronic Hypoparathyroidism: A Meta‐Analysis of Prospective Trials
Source: J Bone Miner Res. 2022 May 20;37(7):1233–50. doi: 10.1002/jbmr.4566 (PMC9545848; doi:10.1002/jbmr.4566)
Supplement: Supplementary file 1 — Appendix S1 Supplementary Information [file JBMR-37-1233-s003.docx]

**Supplementary data**

**Biochemical control**

When available, the supplementary data include for each outcome of interest:

the number of studies reporting the outcome and the relative number of patients (divided by treatment); the baseline and post-treatment weighted averages and standard error (SE) of each parameter; the sensitivity analysis, assuming a correlation of 0.2 or 0.7; where there is no difference in a specific outcome between the two PTH treatments, the mean difference between patients treated with PTH_1-34_ and with PTH_1-84_; funnel plot and/or Egger’s test as appropriate.

In addition: for serum calcium and urinary calcium excretion, subgroup analysis based on oral calcium supplementation regimen, and for serum phosphate and urinary calcium excretion, the mean difference in comparison with the control group.

*Serum calcium*

Data on serum calcium levels before PTH therapy were available in 13 studies on 238 patients (151 taking PTH_1-34_ and 87 taking PTH_1-84_)^(1-13)^. The weighted average at baseline was 2 mmol/l (SE=0.03) in studies on PTH_1-34_ and 2.17 mmol/l (SE=0.05) in studies on PTH_1-84_. Nine studies reported calcium levels after PTH treatment, on a total of 161 patients (104 on PTH_1-34_ and 57 on PTH_1-84_) ^(1-9)^. The weighted average of serum calcium was 2.09 mmol/l (SE=0.06) after PTH_1-34_ and 2.14 mmol/l (SE=0.05) after PTH_1-84_.

Visual inspection of the funnel plot showed a slightly asymmetrical distribution of studies, with most of the studies in the upper side of the funnel (Supplementary Figure 1). However, Egger’s test for asymmetry did not show significant evidence of a small study effect (p=0.283). The asymmetrical distribution may therefore be due to heterogeneity between the studies.

Treatment subgroup analysis for serum calcium levels showed, in patients undergoing PTH_1-34_ therapy, MD of 0.05 mmol/l (95% CI: -0.08 to 0.18 mmol/l; p=0.436), with high heterogeneity (I^2^=94.9%; p<0.001). In contrast, in patients receiving PTH_1-84_ replacement there was a significant reduction in calcium levels (MD=-0.06 mmol/l; 95% CI: -0.10 to -0.03, p=0.001) with non-significant heterogeneity (I^2^= 0%; p=0.592).

Subgroup analysis demonstrated a negative pooled mean difference in patients who completely discontinued supplementation on study entry (MD=-0.06; 95% CI: -0.12 to -0.0001; p=0.049), with a non-significant heterogeneity (I^2^=50.2%; p=0.11), and no difference in the titration group (MD=0.06; 95% CI: -0.08 to 0.203; p=0.411), in which heterogeneity was high (I^2^=95%; p<0.001). In controls, the MD for serum calcium was 0.03 (95% CI: -0.05 to 0.11, p=0.453)^(1,2)^. Sensitivity analysis assuming a correlation of 0.2 or 0.7 confirmed the results.

*Albumin-corrected calcium*

Only 3 studies reported both baseline and post-treatment levels of albumin-corrected calcium and were included in the meta-analysis: 2 on PTH_1-34_ (20 patients)^(14,15)^ and 1 on PTH_1-84_ (49 patients)^(16)^. The overall pooled effect showed a non-significant post-treatment increase (MD=0.95, 95% CI: -0.42 to 2.31; p=0.175). Heterogeneity between the studies was high (I^2^=99.8%; p<0.001). In the PTH_1-34_ subgroup, the increase was significant (MD=0.36; 95% CI: 0.08 to 0.64; p=0.012). Sensitivity analysis assuming a correlation of 0.2 or 0.7 confirmed the results.

*Serum phosphate*

Baseline phosphate levels were available in 14 studies: 11 on PTH_1-34_ (171 patients)^(1-7,11,12,14,15)^ and 3 on PTH_1-84_ (156 patients)^(9,16,17)^. Baseline serum phosphate weighted average was 1.59 mmol/l (SE=0.08) in patients treated with PTH_1-34_ and 1.52 mmol/l (SE=0.02) in patients treated with PTH_1-84_. Data on phosphate levels after PTH treatment were available in 12 studies (9 on PTH_1-34_^(1-3,5-7,11,14,15)^ and 3 on PTH_1-84_^(9,16,17)^) analysing 218 patients (126 after PTH_1-34_ and 92 after PTH_1-84_). Weighted averages were 1.43 mmol/L (SE=0.07) and 1.19 mmol/L (SE=0.1) in patients treated, respectively, with PTH_1-34_ and PTH_1-84_.

The funnel plot inspection showed a symmetrical distribution of the studies, and Egger’s test was not significant (p=0.527) (Supplementary Figure 2). The MD in patients treated with PTH_1-34_ was ‑0.21 mmol/l (95% CI: -0.36 to -0.06 mmol/l; p=0.007), with a high heterogeneity between the studies (I^2^=93.6%; p<0.001). In patients receiving PTH_1-84_, the MD was -0.20 mmol/l (95% CI: -0.24 to -0.16 mmol/l; p<0.001), and the heterogeneity between the studies was high (I^2^=0%; p=0.910). One of the causes of the heterogeneity was the direction of the results; in the study by Winer et al.^(11)^ phosphate levels increased after PTH therapy. The pooled MD in phosphate levels in controls was non-significant (MD=-0.62; 95% CI: -1.67 to 0.43; p=0.249), and heterogeneity between the studies was high (I^2^=99.7%; p<0.001). Sensitivity analysis assuming a correlation of 0.2 or 0.7 confirmed the results.

*Calcium phosphate product*

Baseline calcium phosphate product levels were available in 6 studies (3 on PTH_1-34_^(1,7,15)^ and 3 on PTH_1-84_^(9,16,17)^ including 217 patients (61 under PTH_1-34_ and 156 under PTH_1-84_). The weighted average was 2.76 mmol^2^/l^2^ (SE=0.39) at the baseline and 2.9 mmol^2^/L^2^ (SE: 0.004) post-treatment in studies on PTH_1-34_^(1,7,15)^, and 3.3 mmol^2^/l^2^ (SE=0.06) at the baseline and 2.8 mmol^2^/L^2^ (SE: 0) post-treatment in studies on PTH_1-84_^(9,16,17)^. The asymmetry in the funnel plot upon visual inspection, given the small number of studies, was presumably due to heterogeneity. Sensitivity analysis assuming a correlation of 0.2 or 0.7 confirmed the results.

*25(OH) Vitamin D and 1,25(OH)_2_ Vitamin D*

Visual inspection of studies on 25(OH) vitamin D included in the meta-analysis showed a slight asymmetry of studies towards the bottom right of the funnel plot, but Egger’s test was not significant (p= 0.158).For 25(OH) vitamin D, data from studies on PTH_1-34_ revealed a non-significant pooled reduction (MD=-14.71 pmol/l; 95% CI: -36.52 to 7.11 pmol/l; p=0.186)^(4,11,12,15,18)^. The heterogeneity between the studies was significant (I^2^=93.9%, p<0.001). Data from studies on PTH_1‑84_ also revealed a non-significant pooled reduction (MD=-13.51 pmol/l; 95% CI: -28.15 to 1.12 pmol/l; p=0.070)^(8,9,17,19)^. The heterogeneity between the studies was significant (I^2^=90.6%; p<0.001). Sensitivity analysis assuming a correlation of 0.2 or 0.7 confirmed the results.

For 1,25(OH)_2_ vitamin D, data from studies on PTH_1-34_ showed a non-significant pooled increase (MD=5.23 pmol/l; 95% CI: -11.70 to 22.15 pmol/l; p=0.545) ^(11,12,15,18)^. The heterogeneity between the studies was significant (I^2^=73.6%; p=0.010). Only one study on PTH_1-84_ reported 1,25(OH)_2_ vitamin D levels, and therefore it could not be included in the meta-analysis.

Sensitivity analysis assuming a correlation of 0.2 or 0.7 confirmed the results.

*Urinary calcium excretion*

The funnel plot of studies included in the meta-analysis seemed symmetrical and Egger’s test was not significant (p= 0.241), thus excluding any small-study effect or publication bias (Supplementary Figure 3). The MD in calcium urinary excretion after PTH_1-34_ was non-significant (‑0.89 mmol/24h; 95% CI: -1.93 to 0.15 mmol/24h; p=0.093)^(1-4,7,11,12,14,15)^. In contrast, urinary calcium excretion was significantly reduced after PTH_1-84_ (MD=-1.94 mmol/24h; 95% CI: -2.61 to ‑1.26 mmol/24h; p<0.001)^(8,9,16,17)^. The heterogeneity was high between studies on PTH_1-34_ (I^2^=80.6%; p<0.001), but non-significant between studies on PTH_1-84_ (I^2^=0%; p=0.682).

Subgroup analysis of urinary calcium excretion demonstrated a negative pooled MD in patients who discontinued oral calcium supplementation at study entry (MD=-1.46 mmol/24h; 95% CI: -2.91 to ‑0.020 mmol/24h; p=0.047)^(2-4,11,12)^, with a high heterogeneity (I^2^=83.4%; p<0.001) and no difference in the titration group (MD=-0.56 mmol/24h, 95% CI: -2.30 to 1.17; p=0.411)^(1,7-9)^, with high heterogeneity (I^2^=95%; p<0.001).

The pooled MD in controls under conventional treatment was non-significant (MD=-0.072 mmol/24h; 95% CI: -2.41 to 0.97 mmol/24h; p=0.403). Heterogeneity was high (I^2^=82.1%; p=0.004).

Sensitivity analysis assuming a correlation of 0.2 or 0.7 confirmed the results.

*Subgroup analysis comparing pediatric and adult populations*

We compared the main outcomes by dividing the available data according to the age group of the enrolled patients (pediatric and adult). There was no difference in calcium (p=0.335), phosphate (p=0.638), or urinary calcium excretion (p=0.689).

There seemed to be an age-related difference in 25 OH vitamin D variations: the only study enrolling pediatric patients^(4)^ reported a post-treatment increase, while those studies enrolling adults reported a post-treatment drop in 25 OH Vitamin D.

For calcium-phosphate product, urinary phosphate excretion, bone turnover markers and concomitant calcium salt and active vitamin D supplementations, data were only available for one subgroup or were insufficient to draw any conclusions.

*Subgroup analysis comparing different follow-up durations*

We compared the main outcomes by dividing the available data according to the duration of the study treatment (less than 12 months vs. more than 12 months).

No duration-related difference was found in the mean change from baseline for serum calcium (p=0.227), serum phosphate (p=0.220), calcium-phosphate product (p=0.912) or urinary calcium excretion (p=0.625).

**Bone mineral density (BMD)**

Data on bone mineral density (BMD) obtained by DEXA scan were available in 3 studies on PTH_1-34_^(2,14,15)^. However, in two studies ^(2,15)^ only mean baseline values were provided, therefore the results could not be included in the meta-analysis. In patients treated with PTH_1-84_, data were available at baseline and after PTH replacement in 2 studies (57 patients)^(8,9)^.

*BMD lumbar spine*

The meta-analysis of lumbar spine BMD after PTH_1-84_ showed an increase of 0.045 g/cm^2^ (95% CI: 0.015 to 0.074 g/cm^2^; p=0.003) with non-significant heterogeneity between studies (I^2^=0%; p=0.838). Data on PTH_1-34_ treatment were only available from one study, which reported an MD of 0.07 g/cm^2^ (95% CI: -0.016 to 0.156 g/cm^2^).

*BMD total hip*

The meta-analysis on total hip BMD from studies on PTH_1-84_ revealed an MD of -0.004 g/cm^2^ (95% CI: -0.023 to 0.014 g/cm^2^; p=0.643). Heterogeneity between studies was non-significant (I^2^=0%; p=0.613). The only study available after PTH_1-34_ reported an MD of -0.04 g/cm^2^ (95% CI: -0.12 to 0.03 g/cm^2^). The overall MD was -0.01 (95% CI: -0.025 to 0.011; p=0.473; I^2^=0%, p=0.541).

*BMD femoral neck*

The meta-analysis on femoral neck BMD in studies on PTH_1-84_ also revealed a non-significant variation, with an MD of 0.01 g/cm^2^ (95% CI: -0.005 to 0.033 g/cm^2^; p=0.156). The heterogeneity between the studies was non-significant (I^2^=0%; p=0.923). The only study available after PTH_1-34_ reported an MD of 0 g/cm^2^ (95% CI: -0.039 to 0.039 g/cm^2^). The overall MD was 0.01 g/cm2 (95% CI: -0.006 to 0.028; p=0.202; I^2^=0%, p=0.822).

*BMD distal radius*

The study on PTH_1-34_^(14)^ reported a non-significant variation from baseline (MD=0.030; 95% CI: -0.013 to 0.073), as did the studies on PTH_1-84_^(8,9)^ (MD=-0.010 g/cm^2^; 95% CI: -0.04 to 0.02 g/cm^2^; p=0.517). The heterogeneity between the PTH_1-84_ studies was non-significant (I^2^=68.3% with p=0.076). The overall MD was 0.001 g/cm^2^ (95% CI: -0.027 to 0.025 g/cm^2^; p=0.933; I^2^=60.6%, p=0.079). Sensitivity analysis assuming a correlation of 0.2 or 0.7 confirmed the results.

**Conventional therapy**

*Calcium supplementation*

Data on calcium supplement variations after PTH therapy were available in 12 studies ^(2,3,5-9,14-16,18,20)^. The weighted mean for oral calcium supplementation before PTH therapy was 2.42±0.48 g/day in patients enrolled in trials on PTH_1-34_ (130 patients) and 2.60±0.50 g/day in patients enrolled in trials on PTH_1-84_ (328 patients). After PTH therapy, the weighted mean for calcium supplementation was 0.77±0.17 g/day for PTH_1-34_ treatment (112 patients) and 0.83±0.23 g/day for PTH_1-84_ (106 patients). A funnel plot for studies reporting the change in calcium supplement as absolute values showed symmetrical distribution.

The MD in patients receiving PTH_1-34_ was -1.84 g/day (95% CI: -2.71 to -0.97 g; p<0.001). The heterogeneity between the studies was high (I^2^=95.6%; p<0.001). In patients receiving PTH_1-84_, the MD was -1.37 g/day (95% CI: -1.55 to -1.20 g; p<0.001), and there was no significant heterogeneity between the studies (I^2^=0%; p=0.953).

Data on controls were available from 3 studies^(2,20,21)^. The median of mean calcium supplementation was 1.47 g/day (range 1.00 to 1.93 g/day). Three studies^(2,18,20)^ also reported the mean supplementation at the study end, though data from the study by Winer et al.^(18)^ could not be included due to the protocol design. In the remaining two studies^(2,20)^, there was no reduction in calcium supplementation (0%). Sensitivity analysis assuming a correlation of 0.2 or 0.7 confirmed the results.

*Active vitamin D metabolite supplementation*

Results on the effects of PTH therapy on calcitriol supplementation were extracted from 16 studies^(3,4,6-9,11,12,14-16,18,22-25)^.

The weighted average for calcitriol supplementation before PTH therapy was 0.88 (SE=0.19) μg in patients enrolled in the 9 trials on PTH_1-34_ (124 patients) and 1.19 (SE=0.58) μg in patients enrolled in the 7 trials on PTH_1-84_ (328 patients). The post-therapy weighted average for calcitriol supplementation was 0.13 (SE=0.05) μg for PTH_1-34_ treatment (119 patients), and 0.18 (SE=0.04) μg/daily for PTH_1-84_ (82 patients). Funnel plot for studies reporting the change in calcium supplement as absolute values showed symmetrical distribution.

The meta-analysis of the PTH_1-34_ studies showed a significant MD in calcitriol supplementation of ‑0.62 μg (95% CI: -0.90 to -0.34 μg; p<0.001)^(7,14,15)^, with high heterogeneity between the studies (I^2^=91%; p<0.001). For the PTH_1-84_ studies, there was a significant MD of -0.52 μg (95% CI: -0.62 to -0.42 μg; p<0.001)^(8,16)^ and non-significant heterogeneity between the studies (I^2^=0%; p=0.712).

Data on baseline therapy with active vitamin D metabolites in controls were extracted from 2 studies^(20,21)^. Two other studies provided data on calcitriol supplementation in controls at the end of each trial^(1,2)^ but meta-analysis could not be performed on the type of data provided. Two studies^(20,21)^ provided data on percentage variation in active vitamin D metabolites in controls, showing a non-significant pooled MD of 4% (95% CI: -74% to 83%; p=0.913). The heterogeneity was high (I^2^=98.4%; p<0.001), with one study reporting a 35% reduction ^(21)^ and the other a mean increase of 45%^(20)^ in daily calcitriol dosage in controls. Sensitivity analysis assuming a correlation of 0.2 or 0.7 confirmed the results.

**Supplementary safety analysis**

*Hypocalcaemia*

Hypocalcaemia was reported in 8 studies ^(1,8,9,15,16,20,21,25)^, for a total of 62 of 284 patients (21.8%) (95% CI: 17.2% to 27.1%). This broke down to 6 of 17 (35.3%, 95% CI: 14.2% to 61.7%) patients undergoing PTH_1-34_ and 56 of 267 patients (estimated percentage 20.7%; 95% CI: 16.1% to 26.1%) under PTH_1-84_ with documented hypocalcaemia. Two episodes of hypocalcaemia were considered as treatment-related serious adverse events^(8)^.

*Hypercalcaemia*

Hypercalcaemic episodes were described in 8 studies^(7-9,15,16,20,21,25)^. Seven of these reported the number of patients experiencing hypercalcemia^(8,9,15,16,20,21,25)^, for a total of 56 of 280 patients (20%) (95% CI: 15.5% to 25.2%). Six studies were on PTH_1-84_ ^(8,9,16,20,21,25)^ replacement therapy, with 51 of 270 patients presenting hypercalcemia during PTH therapy. Estimated percentage is 18.9% (95% CI: 14.4% to 24.1%). In the PTH_1-34_ study (15), 5 of 10 patients presented hypercalcemia (50%).

5 studies (1 on PTH_1-34_^(7)^ and 4 on PTH_1-84_^(8,9,20,21)^) also described the number of hypercalcaemic episodes, with a total of 66 episodes, 11 under PTH_1-34_ and 55 under PTH_1-84_. Analysis of the studies that also reported the number of patients with at least one adverse event^(8,9,20,21)^ revealed a total of 55 episodes in 41 patients, an average of 1.3 episode per patient. One hypercalcaemic episode was considered a treatment-related serious adverse event^(21)^.

*Discontinuation*

The estimated percentage of study discontinuation due to PTH-correlated adverse events was 3.7% (95% CI: 1.8% to 6.8%)^(3,6-9,11,14,15,21)^. In 5^(3,8,9,11,14)^ of the 9 studies reporting this result, no patients dropped out due to PTH-related events. When evaluating PTH_1-34_ and PTH_1-84_ therapy separately, discontinuation for therapy-correlated complications occurred in 7.5% (95% CI: 3.5% to 13.8%) of patients taking PTH_1-34_ and in 0.7% (95% CI: 0.02% to 3.7%) of patients taking PTH_1-84_. The PTH-correlated adverse events most commonly leading to study discontinuation were bone pain, nephrolithiasis, depression and headache.

An estimated 2.1% (95% CI: 0.8% to 4.6%) of patients discontinued PTH therapy due to adverse events not correlated with PTH replacement, comprising worsening hypertension and cerebrovascular accident.

*Other adverse events*

For other adverse events, we calculated the percentage of patients affected as summarized in Supplementary Table 1. The paper from Rubin and colleagues^(8)^ provides adverse events as the number of episodes and the percentage of patients for each year of treatment. The most common complaints over the 6 years of the study were musculoskeletal symptoms (95 events), infectious diseases (52 episodes), gastrointestinal symptoms (30 episodes), headache (26 episodes) and nausea (25 episodes).

| Adverse event | Therapy | Study duration  months (median and range) | Number of patients/total | Frequency | References |
| --- | --- | --- | --- | --- | --- |
| Paraesthesia | PTH_1-84_ | 6 (2-60) | 68/237 | 28.7% (23.0-34.9) | ^(9,16,20,21,25)^ |
| Headache | PTH_1-34_ | 3 (3-82.3) | 5/24 | 19.2% (6.6-39.3) | ^(4,15)^ |
|  | PTH_1-84_ | 6 (2-60) | 56/237 | 23.6% (13.2-29.6) | ^(9,16,20,21,25)^ |
| Nausea | PTH_1-34_ | 3.5 | 0/17 | 0% | ^(11)^ |
|  | PTH_1-84_ | 6 (2-60) | 49/237 | 20.7% (15.7 - 26.4) | ^(9,16,20,21,25)^ |
| Hypoaesthesia | PTH_1-84_ | 6 (2-30) | 29/133 | 21.8% (15.1 - 29.8) | ^(9,21,25)^ |
| Diarrhoea | PTH_1-84_ | 45 (30-60) | 21/139 | 15.8% (10.0 - 23.12) | ^(16,21)^ |
| Vomiting | PTH_1-84_ | 45 (30-60) | 17/139 | 12.2% (7.3 - 18.8) | ^(16,21)^ |
| Vertigo | PTH_1-84_ | 30 (6-60) | 18/171 | 10.5% (6.4-16.1) | ^(16,20,21)^ |
| Skin conditions | PTH_1-34_ | 15 (6-24) | 5/50 | 10% (3.3 - 21.8) | ^(12,26)^ |
|  | PTH_1-84_ | 4 (2-6) | 4/74 | 5.4% (1.5-13.3) | ^(20,25)^ |
| Kidney disease | PTH_1-34_ | 24 (3.5-60) | 19/104 | 18.3% (11.4-27.1) | ^(2,7,11,27)^ |
|  | PTH_1-84_ | 6 (2-60) | 13/237 | 5.5% (3.0-9.2) | ^(9,16,20,21,25)^ |
| Arthralgia | PTH_1-84_ | 6 (2-60) | 18/115 | 15.7% (9.5-23.6) | ^(9,16,25)^ |
| Pain in the extremities | PTH_1-34_ | 8.5 (3-36) | 11/60 | 18.3% (9.5- 30.4) | ^(1,2,11,15,18)^ |
|  | PTH_1-84_ | 33 (6-60) | 21/163 | 12.9% (8.2-19.02) | ^(9,16)^ |
| Infections (total) | PTH_1-84_ | 33 (6-60) | 51/80 | 63.8% (52.2-74.2) | ^(16,20)^ |
| Upper respiratory tract infections | PTH_1-34_ | 82.3 | 2/14 | 14% | ^(4)^ |
|  | PTH_1-84_ | 30 (6-60) | 16/171 | 9.9% (4.4-18.5) | ^(16,20,21)^ |
| Common cold | PTH_1-84_ | 33 (6-60) | 16/81 | 19.8% (11.7-30.1) | ^(16,20)^ |
| Urinary tract infections | PTH_1-84_ | 33 (6-60) | 11/81 | 13.6% (7.0-23.0) | ^(16,20)^ |
| Gastrointestinal infections | PTH_1-84_ | 6 (2-60) | 12/123 | 9.8% (5.1-16.4) | ^(16,20,25)^ |
| Flu | PTH_1-84_ | 33 (6-60) | 16/81 | 19.8% (11.7-30.1) | ^(16,20)^ |
| Abdominal pain | PTH_1-84_ | 33 (6-60) | 7/73 | 9.6% (3.9-18.8) | ^(9,16)^ |
| Cardiovascular events | PTH_1-84_ | 30 (6-60) | 18/171 | 10.5% (6.4-16.1) | ^(16,20,21)^ |
| Musculo-skeletal disorders | PTH_1-34_ | 10 | 3/14 | 21.4% | ^(4)^ |
|  | PTH_1-84_ | 6 (6-60) | 64/105 | 60.9% (50.9- 70.3) | ^(9,16,20)^ |
| Hypercalciuria | PTH_1-34_ | 3 | 6/12 | 50% | ^(15)^ |
|  | PTH_1-84_ | 45 (30-60) | 11/139 | 7.9% (4.0-13.7) | ^(16,21)^ |

**Supplementary Table 1. Other side effects described in the trials.**

**References**

1. Winer KK, Sinaii N, Reynolds J, Peterson D, Dowdy K, Cutler GB, Jr. Long-term treatment of 12 children with chronic hypoparathyroidism: a randomized trial comparing synthetic human parathyroid hormone 1-34 versus calcitriol and calcium. J Clin Endocrinol Metab. Jun 2010;95(6):2680-8.

2. Winer KK, Ko CW, Reynolds JC, Dowdy K, Keil M, Peterson D, et al. Long-term treatment of hypoparathyroidism: a randomized controlled study comparing parathyroid hormone-(1-34) versus calcitriol and calcium. J Clin Endocrinol Metab. Sep 2003;88(9):4214-20.

3. Winer KK, Fulton KA, Albert PS, Cutler GB, Jr. Effects of pump versus twice-daily injection delivery of synthetic parathyroid hormone 1-34 in children with severe congenital hypoparathyroidism. J Pediatr. Sep 2014;165(3):556-63 e1.

4. Winer KK, Kelly A, Johns A, Zhang B, Dowdy K, Kim L, et al. Long-Term Parathyroid Hormone 1-34 Replacement Therapy in Children with Hypoparathyroidism. J Pediatr. Dec 2018;203:391-9 e1.

5. Matarazzo P, Tuli G, Fiore L, Mussa A, Feyles F, Peiretti V, et al. Teriparatide (rhPTH) treatment in children with syndromic hypoparathyroidism. J Pediatr Endocrinol Metab. Jan 2014;27(1-2):53-9.

6. Gafni RI, Guthrie LC, Kelly MH, Brillante BA, Christie CM, Reynolds JC, et al. Transient Increased Calcium and Calcitriol Requirements After Discontinuation of Human Synthetic Parathyroid Hormone 1-34 (hPTH 1-34) Replacement Therapy in Hypoparathyroidism. J Bone Miner Res. Nov 2015;30(11):2112-8.

7. Palermo A, Santonati A, Tabacco G, Bosco D, Spada A, Pedone C, et al. PTH(1-34) for Surgical Hypoparathyroidism: A 2-Year Prospective, Open-Label Investigation of Efficacy and Quality of Life. J Clin Endocrinol Metab. Jan 1 2018;103(1):271-80.

8. Rubin MR, Cusano NE, Fan WW, Delgado Y, Zhang C, Costa AG, et al. Therapy of Hypoparathyroidism With PTH(1-84): A Prospective Six Year Investigation of Efficacy and Safety. J Clin Endocrinol Metab. Jul 2016;101(7):2742-50.

9. Lakatos P, Bajnok L, Lagast H, Valkusz Z. An Open-Label Extension Study of Parathyroid Hormone Rhpth(1-84) in Adults with Hypoparathyroidism. Endocr Pract. May 2016;22(5):523-32.

10. Misof BM, Roschger P, Dempster DW, Zhou H, Bilezikian JP, Klaushofer K, et al. PTH(1-84) Administration in Hypoparathyroidism Transiently Reduces Bone Matrix Mineralization. J Bone Miner Res. Jan 2016;31(1):180-9.

11. Winer KK, Yanovski JA, Sarani B, Cutler GB, Jr. A randomized, cross-over trial of once-daily versus twice-daily parathyroid hormone 1-34 in treatment of hypoparathyroidism. J Clin Endocrinol Metab. Oct 1998;83(10):3480-6.

12. Winer KK, Zhang B, Shrader JA, Peterson D, Smith M, Albert PS, et al. Synthetic human parathyroid hormone 1-34 replacement therapy: a randomized crossover trial comparing pump versus injections in the treatment of chronic hypoparathyroidism. J Clin Endocrinol Metab. Feb 2012;97(2):391-9.

13. Winer KK, Sinaii N, Peterson D, Sainz B, Jr., Cutler GB, Jr. Effects of once versus twice-daily parathyroid hormone 1-34 therapy in children with hypoparathyroidism. J Clin Endocrinol Metab. Sep 2008;93(9):3389-95.

14. Upreti V, Somani S, Kotwal N. Efficacy of Teriparatide in Patients with Hypoparathyroidism: A Prospective, Open-label Study. Indian J Endocrinol Metab. May-Jun 2017;21(3):415-8.

15. Marcucci G, Masi L, Cianferotti L, Giusti F, Fossi C, Parri S, et al. Chronic hypoparathyroidism and treatment with teriparatide. Endocrine. Feb 4 2021.

16. Mannstadt M, Clarke BL, Bilezikian JP, Bone H, Denham D, Levine MA, et al. Safety and Efficacy of 5 Years of Treatment With Recombinant Human Parathyroid Hormone in Adults With Hypoparathyroidism. J Clin Endocrinol Metab. Nov 1 2019;104(11):5136-47.

17. Clarke BL, Vokes TJ, Bilezikian JP, Shoback DM, Lagast H, Mannstadt M. Effects of parathyroid hormone rhPTH(1-84) on phosphate homeostasis and vitamin D metabolism in hypoparathyroidism: REPLACE phase 3 study. Endocrine. Jan 2017;55(1):273-82.

18. Winer KK, Yanovski JA, Cutler GB, Jr. Synthetic human parathyroid hormone 1-34 vs calcitriol and calcium in the treatment of hypoparathyroidism. JAMA. Aug 28 1996;276(8):631-6.

19. Sikjaer T, Rolighed L, Hess A, Fuglsang-Frederiksen A, Mosekilde L, Rejnmark L. Effects of PTH(1-84) therapy on muscle function and quality of life in hypoparathyroidism: results from a randomized controlled trial. Osteoporos Int. Jun 2014;25(6):1717-26.

20. Sikjaer T, Rejnmark L, Rolighed L, Heickendorff L, Mosekilde L, Hypoparathyroid Study G. The effect of adding PTH(1-84) to conventional treatment of hypoparathyroidism: a randomized, placebo-controlled study. J Bone Miner Res. Oct 2011;26(10):2358-70.

21. Mannstadt M, Clarke BL, Vokes T, Brandi ML, Ranganath L, Fraser WD, et al. Efficacy and safety of recombinant human parathyroid hormone (1-84) in hypoparathyroidism (REPLACE): a double-blind, placebo-controlled, randomised, phase 3 study. Lancet Diabetes Endocrinol. Dec 2013;1(4):275-83.

22. Harslof T, Sikjaer T, Sorensen L, Pedersen SB, Mosekilde L, Langdahl BL, et al. The Effect of Treatment With PTH on Undercarboxylated Osteocalcin and Energy Metabolism in Hypoparathyroidism. J Clin Endocrinol Metab. Jul 2015;100(7):2758-62.

23. Vokes TJ, Mannstadt M, Levine MA, Clarke BL, Lakatos P, Chen K, et al. Recombinant Human Parathyroid Hormone Effect on Health-Related Quality of Life in Adults With Chronic Hypoparathyroidism. J Clin Endocrinol Metab. Feb 1 2018;103(2):722-31.

24. Cusano NE, Rubin MR, McMahon DJ, Irani D, Anderson L, Levy E, et al. PTH(1-84) is associated with improved quality of life in hypoparathyroidism through 5 years of therapy. J Clin Endocrinol Metab. Oct 2014;99(10):3694-9.

25. Bilezikian JP, Clarke BL, Mannstadt M, Rothman J, Vokes T, Lee HM, et al. Safety and Efficacy of Recombinant Human Parathyroid Hormone in Adults With Hypoparathyroidism Randomly Assigned to Receive Fixed 25-mug or 50-mug Daily Doses. Clin Ther. Oct 2017;39(10):2096-102.

26. Santonati A, Palermo A, Maddaloni E, Bosco D, Spada A, Grimaldi F, et al. PTH(1-34) for Surgical Hypoparathyroidism: A Prospective, Open-Label Investigation of Efficacy and Quality of Life. J Clin Endocrinol Metab. Sep 2015;100(9):3590-7.

27. Gafni RI, Langman CB, Guthrie LC, Brillante BA, James R, Yovetich NA, et al. Hypocitraturia Is an Untoward Side Effect of Synthetic Human Parathyroid Hormone (hPTH) 1-34 Therapy in Hypoparathyroidism That May Increase Renal Morbidity. J Bone Miner Res. Oct 2018;33(10):1741-7.

**Supplementary Figures - Legend**

**Supplementary Figure 1.** Funnel plot for distribution of studies on changes in serum calcium in patients treated by PTH 1-34 and controls.

**Supplementary Figure 2.** Funnel plot for distribution of studies on changes in serum phosphate levels in patients treated by PTH 1-34 and controls.

**Supplementary Figure 3.** Funnel plot for distribution of studies on changes in 24h urinary calcium excretion levels in patients treated by PTH 1-34 and controls.

**Supplementary Figure 4.** Bubble chart of percentage of reduction in calcium supplementation after PTH 1-34 (dark grey) and PTH 1-84 (light grey) treatment. Trials in which discontinuation of conventional therapy was not titrated according to serum calcium are striped.

**Supplementary Figure 5.** Bubble chart of percentage of reduction in calcitriol supplementation after PTH 1-34 (dark grey) and PTH 1-84 (light grey) treatment. Trials in which discontinuation of conventional therapy was not titrated according to serum calcium are striped.
