# Supplementary figures and images for: Safety and Efficacy of PTH 1‐34 and 1‐84 Therapy in Chronic Hypoparathyroidism: A Meta‐Analysis of Prospective Trials
Source: J Bone Miner Res. 2022 May 20;37(7):1233–50. doi: 10.1002/jbmr.4566 (PMC9545848; doi:10.1002/jbmr.4566)

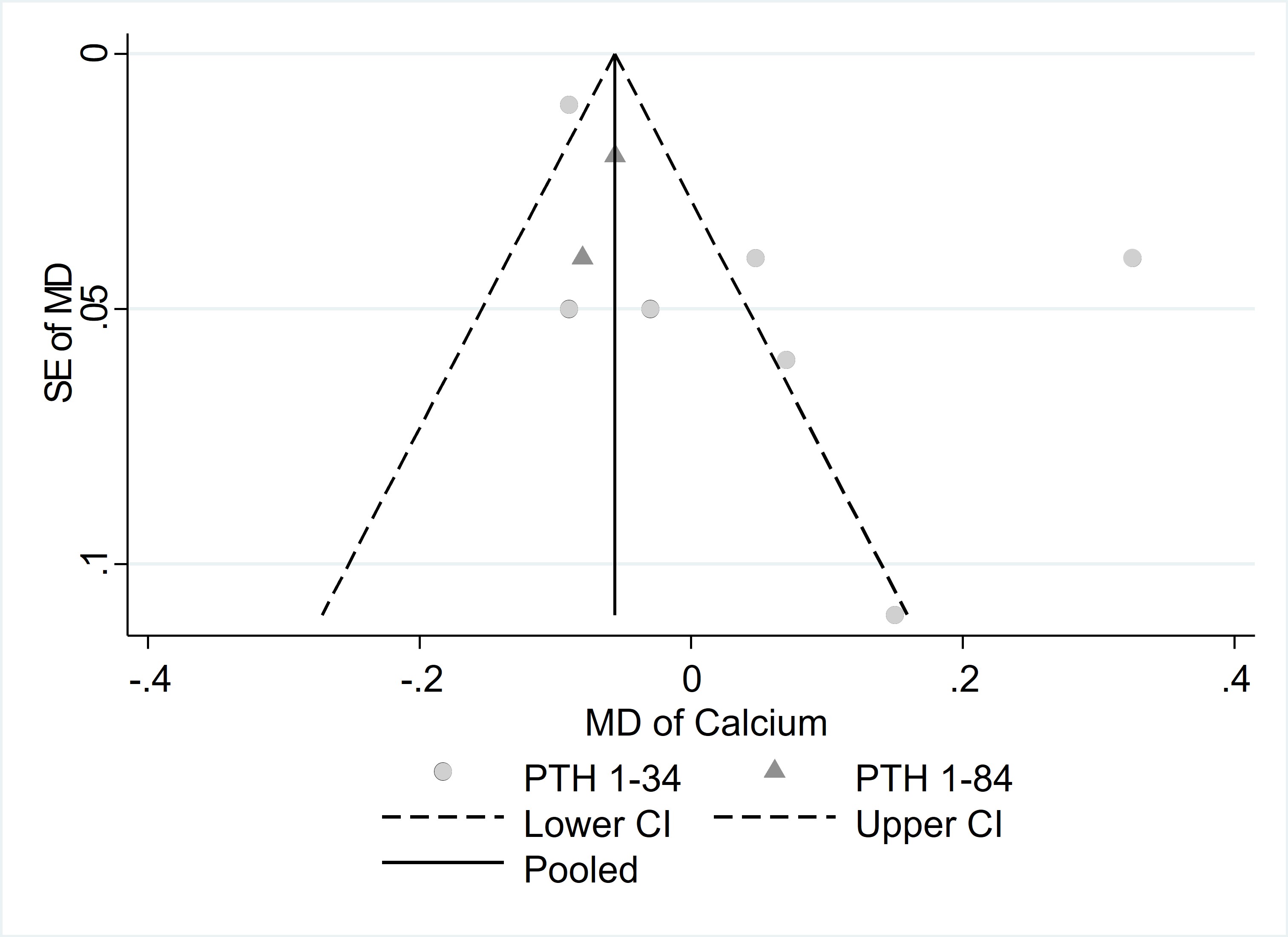

Supplement: Supplementary file 2 — Supplementary Fig. S1 Funnel plot for distribution of studies on changes in serum calcium in patients treated by PTH 1‐34 and controls. [file JBMR-37-1233-s002.tif]

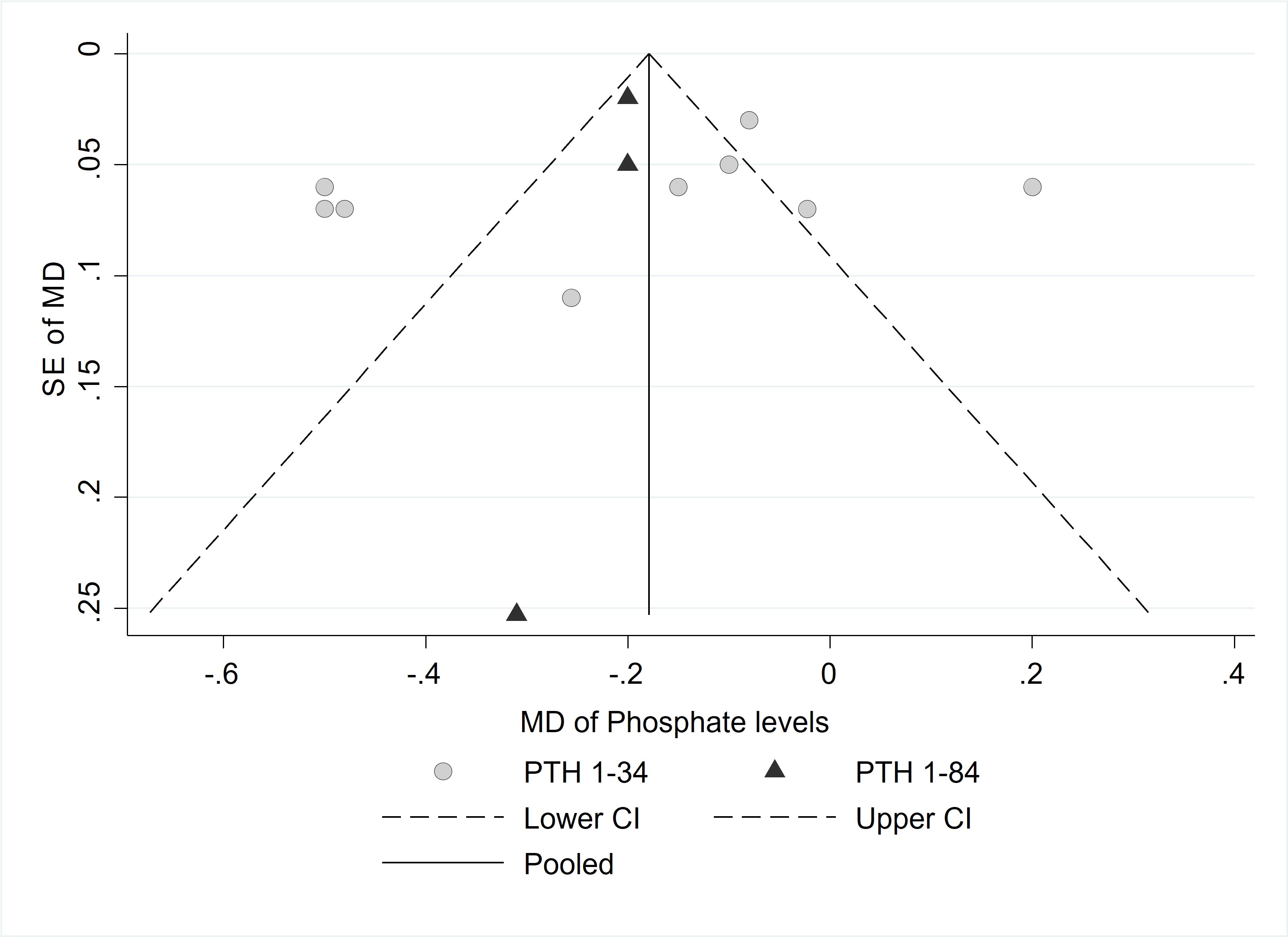

Supplement: Supplementary file 3 — Supplementary Fig. S2 Funnel plot for distribution of studies on changes in serum phosphate levels in patients treated by PTH 1‐34 and controls. [file JBMR-37-1233-s006.tif]

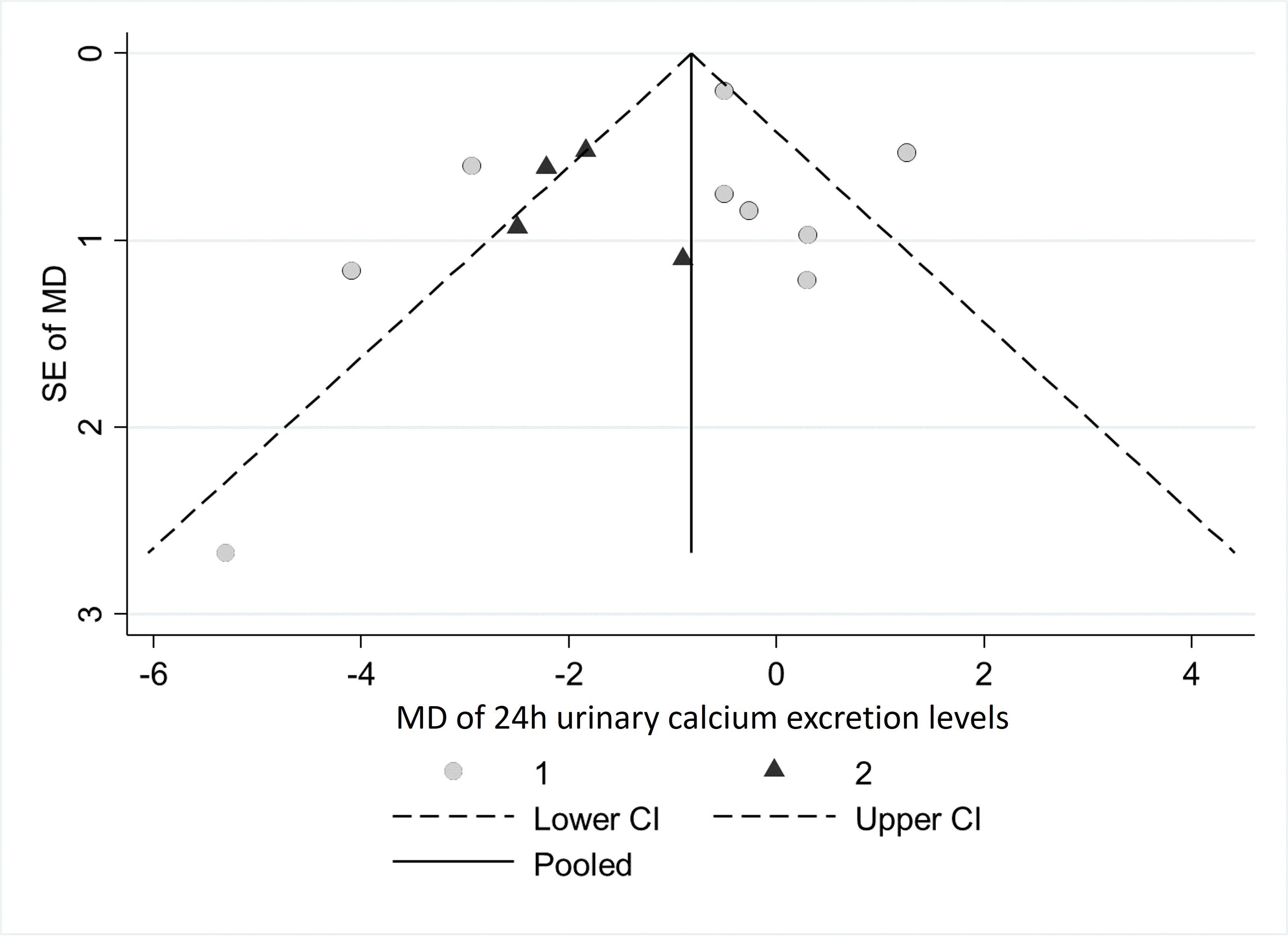

Supplement: Supplementary file 4 — Supplementary Fig. S3 Funnel plot for distribution of studies on changes in 24‐hour urinary calcium excretion levels in patients treated by PTH 1‐34 and controls. [file JBMR-37-1233-s005.tif]

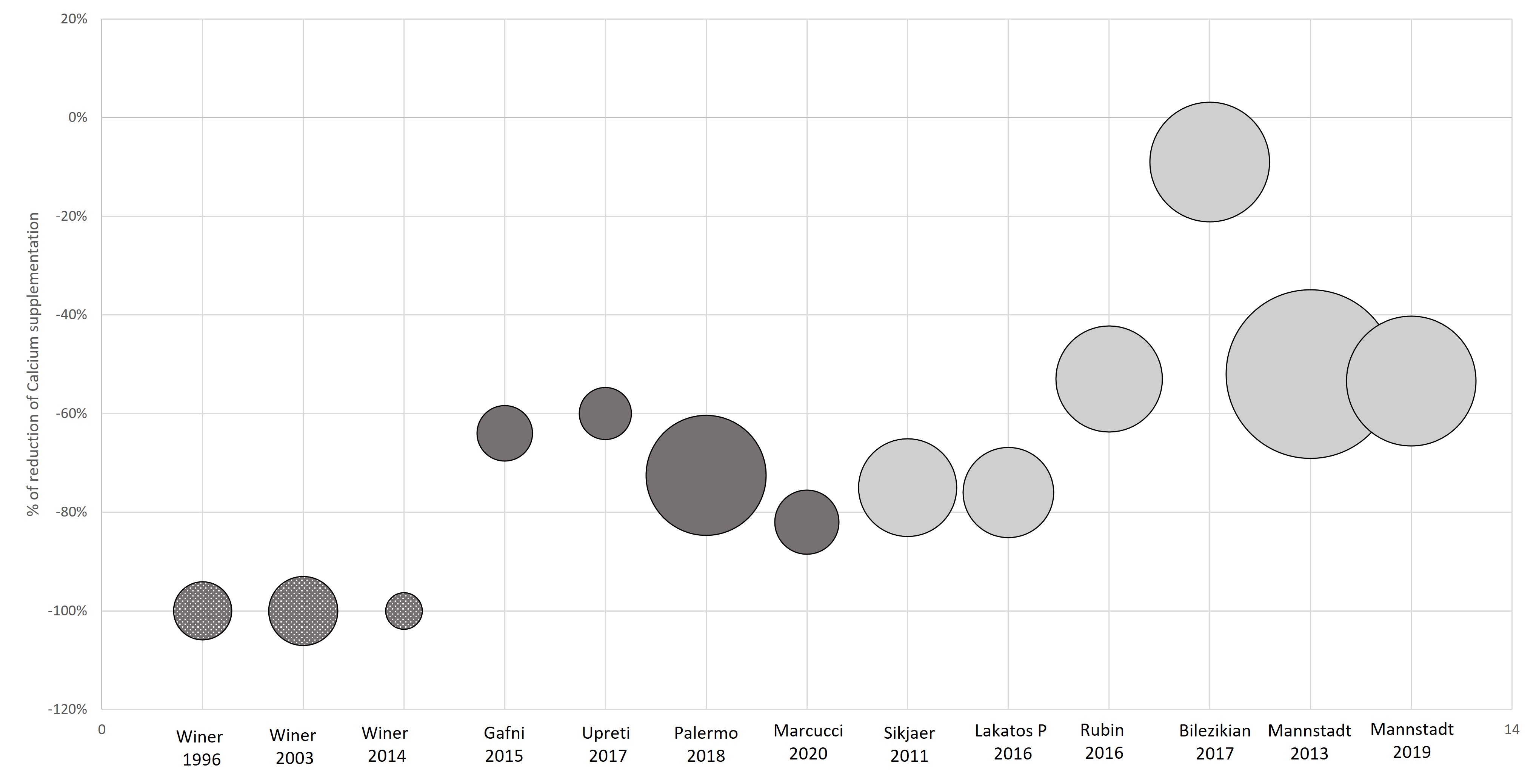

Supplement: Supplementary file 5 — Supplementary Fig. S4 Bubble chart of percentage of reduction in calcium supplementation after PTH 1‐34 (dark gray) and PTH 1‐84 (light gray) treatment. Trials in which discontinuation of conventional therapy was not titrated according to serum calcium are striped. [file JBMR-37-1233-s004.tif]

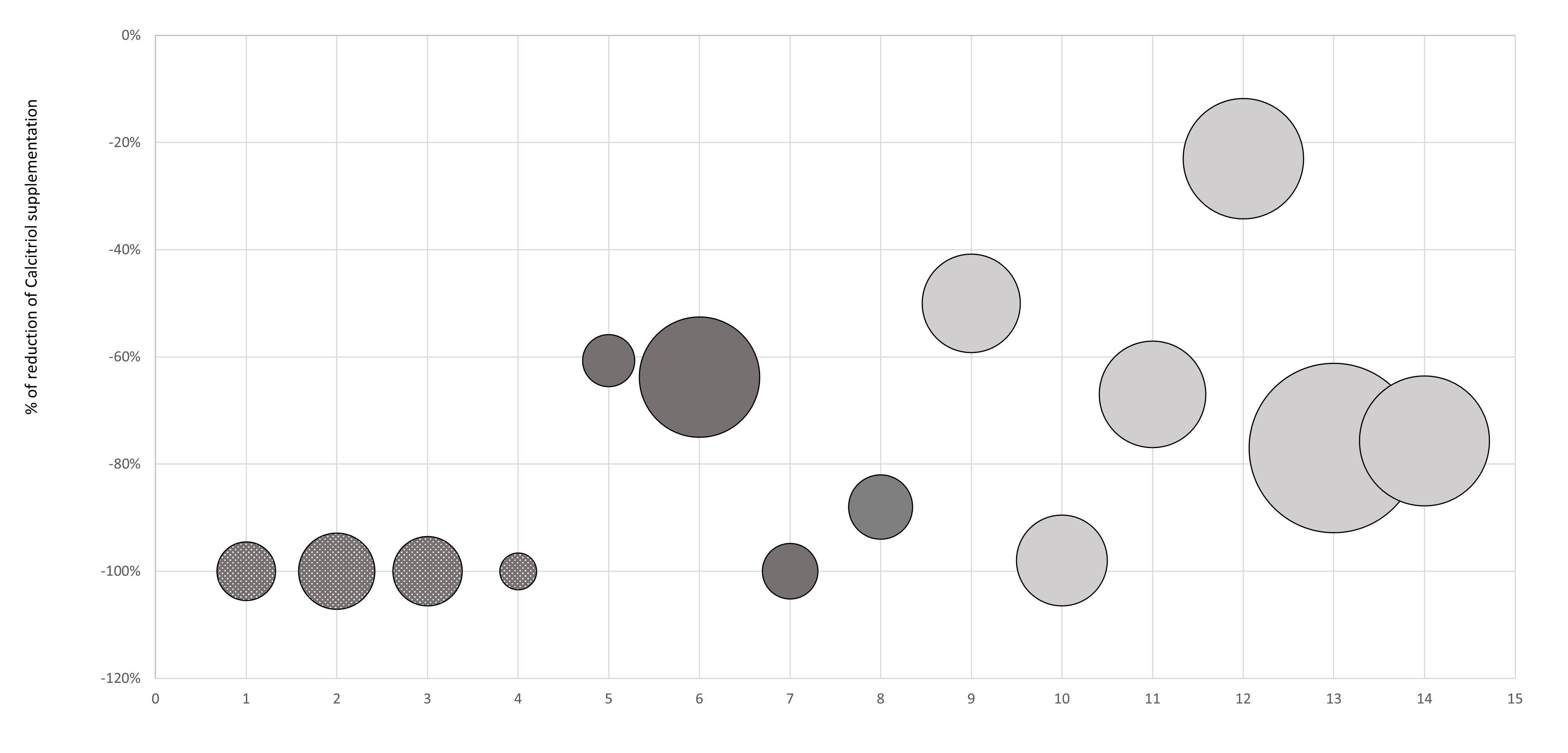

Supplement: Supplementary file 6 — Supplementary Fig. S5 Bubble chart of percentage of reduction in calcitriol supplementation after PTH 1‐34 (dark gray) and PTH 1‐84 (light gray) treatment. Trials in which discontinuation of conventional therapy was not titrated according to serum calcium are striped. [file JBMR-37-1233-s001.tif]
